# Sub-cellular internalization and organ specific oral elivery of PABA nanoparticles by side chain variation

**DOI:** 10.1186/1477-3155-9-10

**Published:** 2011-03-28

**Authors:** Jhillu S Yadav, Pragna P Das, T Lakshminarayan Reddy, Indira Bag, Priyadarshini M Lavanya, Bulusu Jagannadh, Debendra K Mohapatra, Manika Pal Bhadra, Utpal Bhadra

**Affiliations:** 1 Indian Institute of Chemical Technology Uppal Road, Hyderbad-500007, India; 2 Centre for Cellular and Molecular Biology, Uppal Road, Hyderabad 500007, India

## Abstract

**Background:**

Organic nanomaterials having specific biological properties play important roles in *in vivo *delivery and clearance from the live cells. To develop orally deliverable nanomaterials for different biological applications, we have synthesized several fluorescently labelled, self-assembled PABA nanoparticles using possible acid side chain combinations and tested against insect and human cell lines and *in vivo *animal model. Flurophores attached to nanostructures help in rapid *in vivo *screening and tracking through complex tissues. The sub-cellular internalization mechanism of the conjugates was determined. A set of physio-chemical parameters of engineered nanoskeletons were also defined that is critical for preferred uptake in multiple organs of live *Drosophila*.

**Results:**

The variability of side chains alter size, shape and surface texture of each nanomaterial that lead to differential uptake in human and insect cells and to different internal organs in live *Drosophila *via energy dependent endocytosis. Our results showed that physical and chemical properties of C-11 and C-16 acid chain are best fitted for delivery to complex organs in *Drosophila*. However a distinct difference in uptake of same nanoparticle in human and insect cells postulated that different host cell physiology plays a critical role in the uptake mechanism.

**Conclusions:**

The physical and chemical properties of the nanoparticle produced by variation in the acid side chains that modify size and shape of engineered nanostructure and their interplay with host cell physiology might be the major criteria for their differential uptake to different internal organs.

## Background

Integration of nanostructure with biomolecules, biosensors and drugs has established a strong framework for advancements in medical diagnostics, therapeutics and hold enormous promises for bioengineering applications [1,2]. In recent years, a wide variety of inorganic nanomaterials with distinct shapes and sizes (for example nanoparticles, nanorods, nanowires, nanofibres and nanotubes) have been used as delivery vehicles [3-5]. But two major issues i.e., targeted release of the biomolecules and rapid clearance of the carriers that are considered for delivery in live cells still remain unanswered [6]. It has led to the failure of many inorganic nanostructures as attractive vehicles [7,8] and has opened a window of opportunity for the development of nanoparticles from organic materials. These nanomaterials are well accepted in bio-systems because they hold more chemical flexibility, surface configuration better tissue recognition and cell uptake ability [9].

In general, basic cell physiology and cell surveillance do not allow easy accessibility of foreign particles inside the cells. Exhaustive efforts are being carried out for engineering smooth delivery vehicles, synthesized from biocompatible and biodegradable materials. Though use of nano-materials has been successful in *in vitro *cultured cells [10], in practice, its adaptability in *in vivo *organ tracking by repeated injections is more challenging because of its limited self-life, delivery hurdles, and compatibility to fragile cell environment and potent immunogenicity [11]. Major improvements on chemical modifications of nano-materials play a fundamental role in cell uptake and live tissue distribution [12]. The surface texture by using small molecules, side chains and other conjugates alter the biological properties of nano cargoes [13]. We therefore hypothesized that such variation could increase smooth transition to shuttle inside live cells. To date, efforts for surface modifications of organic nanostructures have been rare. It is mainly due to lack of self-assembled organic molecules and compatibility of small molecules with nanoskeleton [14-16].

A handful of organic nanomaterials are presently known to cross cell membrane barriers for delivery of biological agents [15,16]. Our previous studies showed that long chain alkyl 4-N-pyridin-2-yl-benzamides are capable of "bottom-up" self-assembly to furnish nanomaterials and accomplish oral delivery in *in vivo *models [12]. Though earlier we have established that PABA conjugates shuttle inside the cells and serve as ideal cargo for delivery in model organism *Drosophila *[12] the detailed parameters for cellular uptake mechanism and pathway of entry was still missing. Moreover, it is critical to know whether the variation of side chains in PABA conjugates have any impact on cellular internalization mechanism and targeting to internal organs in *in vivo *models. Here we used *p*-aminobenzoic acid (PABA) as skeletal moiety and self assembled with different acid side chains to produce a library of fluorescent organic PABA nano-particles having different shapes and sizes and determined their mode of live cell entry. We identified nanoparticles that discriminate among different physiological environments of human cells and insect cells. Simultaneously, we observed many physico-chemical properties of PABA nanoparticles and their uptake mechanism that facilitates targeted organ delivery via oral consumption.

## Results

### Synthesis of nanoparticles

Nanoparticles with different side chain variations were synthesized (Additional File 1).

**Figure 1:**
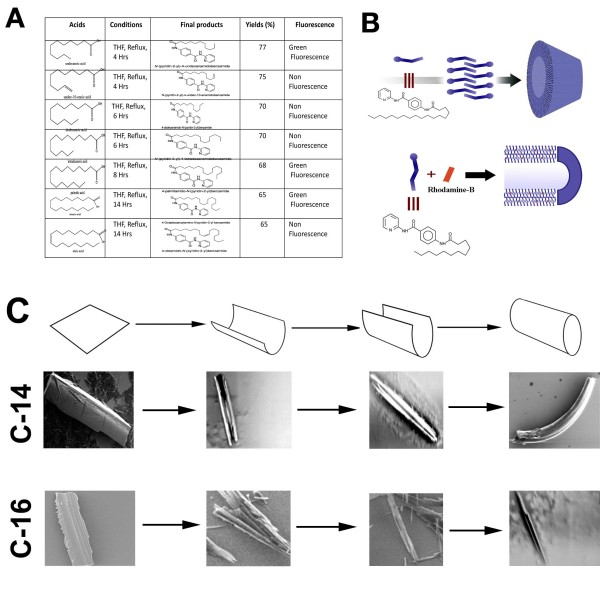
**Design and synthesis of nanomaterials**. (A) Chemical structure of acid side chains, final self assembled product reaction condition, percentage of yield, fluorescent dyes summarized in a table. (B) Schematic diagram showing formation of two nanoparticles (C12 and C18) was shown (C) cartoon diagram and compatible SEM images showing rollover mechanism of two nanomaterial (C-14 and C16) formation.

The synthesis involved amide formation with 2-aminopyridine followed by reduction of the nitro functionality using Pd/C under hydrogen atmosphere as the reducing agent. The free amine functionality present in benzamide was coupled with different acid chlorides as depicted above. Only seven compounds were subjected to self-assembly of conjugated nanoparticle formation (Figure 1A). To obtain self-assembled nanostructure in each case, 1 mg compound (1-7) was added to 2 mL methanol and heated at 60°C till it dissolved completely. Subsequently, 2 mL deionised water was mixed slowly at the same temperature to obtain a pure white solution, which on slow cooling at room temperature formed cotton dust-like white aggregates (Additional File 2). These aggregates were isolated using centrifuge at 4,500 rpm for 20 min, followed by overnight drying at 60°C to afford 0.5 mg of final nano-materials (Additional File 2 Figure S1-10). The PABA nanomaterials thus obtained from compound 1-7 were named as C-11, C-11U, C-12, C-14, C-16, C-18, C-18U respectively, based on the length of the side chains and unsaturated moieties coupled during synthesis (Figure 1A).

It is important to understand the self-assembled procedure and the size and shape of different nanoparticles biophysically. Though, the exact mechanism of self assembly is still not clear, we believe that hydrogen bonded aggregates were formed with limited motion of the molecules. The self-assembly occurred due to arrangement of the molecules in stack and thereby allowing the transition to the lower couple excited state of the molecules, which favours the enhancement of the emission (Figure 1B-C).

### Characterisation of nanoparticles: Laser confocal and scanning electron microscopy

**Figure 2:**
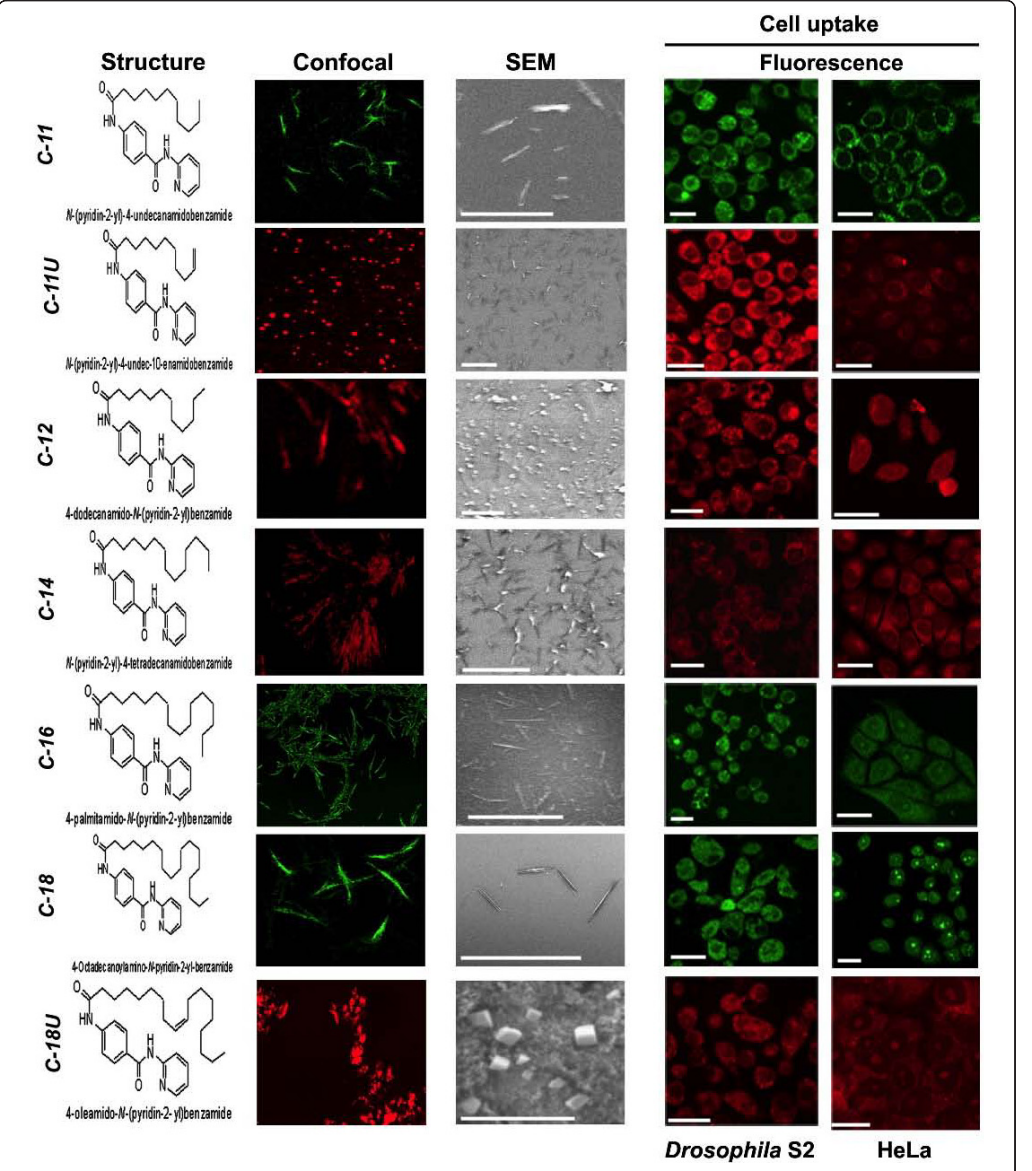
**Physico-chemical properties and microscopic views of seven PABA anomaterials**. elative Uptake of several nanomaterials in insect (*Drosophila *S2) and human tumour cells (HeLa) were shown. The differences in chemical structure, shape and surface texture of nanomaterials leads to a variation in cell uptake. Scale- 250 nm (SEM), 50 μm (cells).

Laser confocal microscopic images showed that three nanostructures, C-11, C-16 and C-18 emitted intrinsic green fluorescence, while remaining four nanomaterials (C-11U, C-12, C-14, C-18U) did not emit any intrinsic fluorescence (Figure 2). For their *in vitro *and *in vivo *tracking, these nano structures were prepared by embedding rhodamine-B to the nano walls. Rhodamine B solution (0.1 mL, 1 mg of Rhodamine B in 5.0 mL of deionized water) was added prior to the addition of deionized water (2 mL) which, on slow cooling, produced pink-coloured aggregates. These were isolated and dried following same experimental condition as noted above (Figure 1B).

To verify the fluorescence enhancement, induced by self-assembly nanostructure, the fluorescence emission of the monomer and the self-assembled nanoparticles were compared using Nanodrop 3300 fluoro-spectrometer. The fluorescence intensity of the nanostructures (determined by a methanol/water solution) using blue diode option (maximum excitation 477 nm) was much stronger and found in 510 nm than that of the non-fluorescent monomer (studied in CH_2_Cl_2_, where it does not aggregate) under the same 0.3 wt % concentration. Laser confocal and scanning electron microscopic (SEM) images showed that the shape and size of each self assembled benzamide structure differs based on the length of the acid side chain (Figure 2).

The saturated acid side chains mainly form tubular shape structure with a hollow space inside, while unsaturated acid chlorides produced cube shaped particles (Figure 2). It also appears that 4-alkylamido-*N*-pyridin-2yl-benzamides when conjugated with saturated acid chlorides forms sheet like structures initially. The folding of the extended sheets along one axis leads to the formation of the nanotubular structures in solution [17]. TEM and SEM images of half tubes and tubular structure with hollow space inside support the model (Figure 1C).

### Dynamic light scattering study

To ascertain the size, a Dynamic Light-Scattering (DLS) study was carried out using different nanoparticles produced by side chain variation. In all cases, freshly prepared nanomaterials were mostly uniform in size with very few submicron sized aggregates, while materials examined after prolonged storage (after 3 days) contains more micron sized aggregates. DLS studies from fresh preparations estimated an average size in the range of 100 to 200 nm but prolonged storage leads to the formation of submicron-sized structures (Additional File 2 Figure S11). The average height of each nanoparticle as measured by 3 D reconstituted AFM images is 3-5 nm.

### Relative uptake of nanomaterials in insect and human cell lines

All nanoparticles preserve the biological properties of PABA in self-assembled conjugates as monitored by the growth and viability of the wild type bacterial strains (*E. coli *K12) in cultured media in the presence of PABA or PABA containing nanostructures. Nearly an equal level of bacterial growth in culture media containing PABA or PABA nanomaterials revealed that PABA properties are still intact in PABA conjugated nanomaterials.

To screen the relative uptake of the nanoparticles in cross species cell lines (insect and human) *in vitro *and also to estimate the accumulated nanomaterials inside the subcellular organelles, three different cell lines *Drosophila *S2 (Figure 2), neoplastic HeLa cells and nonneoplastic Human Embryonic Kidney (HEK-293) (Figure 2; Additional File 2 Figure S12) were cultured in media containing different concentrations of all the nanomaterial; 10 μg/ml, 30 μg/ml and 60 μg/ml in 0.01% DMSO. In all cases, nanomaterial containing media to a final concentration 60 μg/ml in 0.01% DMSO showed no adverse effect on cell physiology. Accumulation of nanomaterials varied widely based on the side chains of PABA conjugates inside both insect (*Drosophila *S2) and human (HEK293, HeLa) cells (Figure 2; Additional File 2 Figure S12). Indeed, nanoparticles that emit green fluorescence (C-11, C-16 and C-18) accumulate almost equally in all three cell types despite the differences in the length of carbon side chains. These results suggest that the tubular shape of all three nanostructures is more important than the length of the acid chains for cell entry. The accumulation increased proportionately to the concentration of incubated nanoparticles and time. Moreover, uptake of C-12 and C-14 having nearly identical shape, are more intense relative to unsaturated acid chains (C-11U and C-18U) in human cells. It is possible that chemical properties of the unsaturated side chain might hinder the cellular entry. In contrast, a distinct internal cell environment of *Drosophila *S2 cells increase the uptake of unsaturated C-11U particles. These results demonstrated that three major factors; shape, properties associated with unsaturated side chain and cross species cell physiology are involved in the rate of cellular uptake. (Figure 2; Additional File 2 Figure S12).

Since rhodamine was not covalently bonded with nanostructure, we cannot rule out the possibility that rhodamine might be released from the nano walls during cell uptake. To eliminate such possibility, we incubated both the insect and human cells with rhodamine dye as well as rhodamine intercalated nanomaterials (C-11U and C-14) separately under same experimental conditions. After equal period of incubation, cells from both conditions were processed and viewed under confocal microscope. Cells cultured with only rhodamine showed accumulation at the outer periphery with negligible amount inside, while an intense fluorescence was seen inside the cells cultured with rhodamine containing nanoparticles indicating that rhodamine dye was intact in the nanostructure (Additional File 2 Figure S13).

### Effect of nanoparticles on cell viability and cytotoxicity

**Figure 3:**
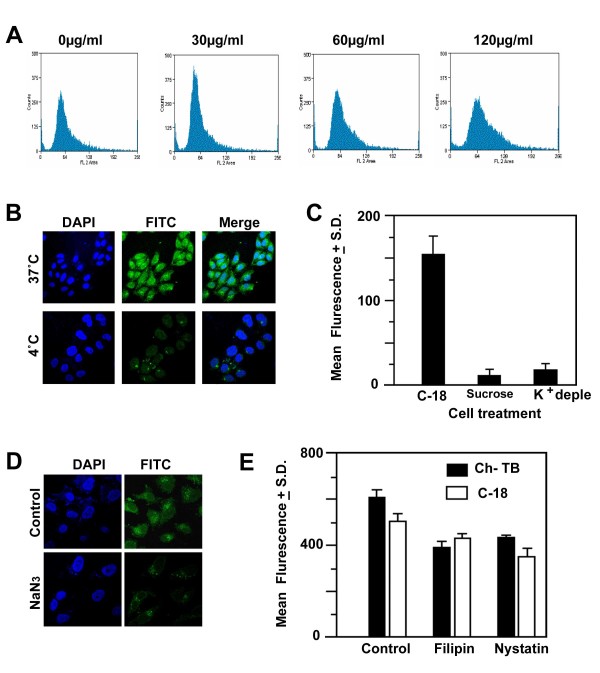
**Sub-cellular internalization of nanomaterials in human cultured cells**. (A) Cell-cycle arrest and cell viability were tested by Flow cell cytometry data of HEK-293 cells obtained after incubation in culture media containing different concentration of nanoparticles C-16. (B) The confocal images of HeLa cells after incubation at 37°C and 4°C in nanoparticles (C-16 containing media) (C) cells pre-treated with 0.45 M sucrose and K^+ ^- depleted medium, (D) after pre-treatment with NaN3 respectively (E) Flow cytometry data of HeLa cells with no pre-treatment and pre-treated with filipin and nystatin were presented in a bar diagram. Cholera toxin B (Black) and C-18 nanoparticle (blank) uptake was shown. Scale 50 μm (Cells).

To address cell viability and cyto-toxicity, colorimetric assay was performed using 3-(4-5-dimethylthiazol-2-yl)-2,5-diphenyltetrazoliumbromide. The cells incubated in freshly prepared nanoparticles containing media were treated with MTT. Uptake of nanoparticles in all cell types does not disturb normal cell propagation and showed more than 90% cell viability even at the higher nanomaterial concentration (120 μg/ml) relative to DMSO treated cells (Additional File 2 Figure S14). These results suggest that nanomaterials function as efficient bio-transporters and fail to show any cytotoxicity. These findings were further verified by a parallel study using flow-cytometry measurements. The mitotic cells from confluent cultures incubated with different nanomaterials containing media were monitored. The relative progression of cells from G1 to S phase was also determined. In three separate cultures containing 0 μg/ml, 30 μg/ml, 60 μg/ml nanoparticles, the phases of cell cycles were progressing normally based on the incubation time, but in higher concentration (120 μg/ml), a fall of G1 number with concurrent increase in G2 and S phase was noticed indicating progression towards asynchrony (Figure 3A; Additional File 2 Figure S15).

### Mode of uptake of PABA nanomaterials

Broadly, there are two mode of entries, either PABA nanomaterials transverse the cell membrane via endocytosis or energy independent nonendocytotic mechanism. We have carried out a series of investigations on uptake mechanism and cellular internalization for PABA conjugates. Endocytosis is an energy dependent mechanism. The process is hindered at a low temperature (at 4°C instead of 37°C) or in ATP deficient environment. Cells incubated in media containing nanoparticles were either cultured at 4°C or pretreated with NaN3 for inhibiting the production of ATP, thereby hampering the endocytosis process. The level of fluorescent intensity in the cytosol of each cultured cells was reduced dramatically relative to cells cultured in regular standard conditions (Figure 3B, D). This reduction determine that PABA conjugates enter in the sub-cellular compartment of cultured cells via endocytosis.

We also sub-categorized the endocytosis pathway including phagocytosis, pinocytosis, clathrin dependent receptor mediated and clathrin independent mechanisms. Internalization often occurs when the clathrin coat on the plasma membrane forms conspicuous invagination in the cell membrane leading to the budding of clathrin-coated vesicles. As a result, extracellular species located on the cell membrane are trapped within the vesicles and invaginated inside the cells [18,19]. To disrupt the formation of clathrin coated vesicles on the cell membranes, cells were preincubated in sucrose (hypertonic) soluton or K^+^-depleted media before treatment with all seven nanoparticles. Data showed a drastic reduction in PABA nanoparticle uptake (Figure 3C), which suggests that a clathrin dependent endocytosis process is involved in entry mechanism.

### Uptake of PABA nanomaterials by clathrin dependent endocytosis

To rule out the possibility of cellular uptake of PABA conjugates via caveolae or lipid rafts pathway, we pretreated the cells with drug filipin and nystatin, which disrupt cholesterol distribution within the cell membrane [20]. In contrast to clathrin blocking experiments, pretreatment of the drugs had a negligible blockage on the cellular uptake, which suggests a little or no involvement of the caveolae dependent cell entry. In a similar control experiment we studied the uptake of fluroscent labelled cholera toxin B (CTX-B) which is a multivalent ligand protein known to be internalised by caveolae depenent endocytic pathway (Figure 3E). The CTX-B showed a significant inhibition in cell entry in the presence of filipin and nystatin. Taken together, the results verify that cellular internalization of PABA conjugates is mediated through the clathrin-dependent endocytosis pathway.

### Oral uptake of variable PABA nanomaterials in *Drosophila*

Organic nano-assemblies have negligible adverse effect on cellular physiology, behaviour, sensitivity to adult sex and other pharmacokinetics parameters of *Drosophila*. We have screened nanoparticles conjugated with variable side chains for organ specific targeting in *Drosophila *[20,21]. Different sets of larvae, pupae and adult flies were grown with sole feeding of nanoparticle containing media. The accumulation to various tissues, selective organ uptake and their clearance was also monitored by imaging the fluorescence signals during the stages of development in *Drosophila*. In live insects, oral feeding of nanomaterials causes systemic spreading of signals through the gut by peristaltic movement to cross the cell membrane barrier. In general, majority of the nanoparticles carrying unsaturated side chains (C-11U, C-18U) showed a low level of incorporation in all stages of *Drosophila *life cycle although C-18U showed a comparatively high level of incorporation in two different life stages, larvae and pupae (Additional File 2 Figure S16A-B). We further investigated the efficacy of *in vivo *targeted delivery among nanoparticles that emits intrinsic green and nanoparticles with intercalated rhodamine B in the wall. Intrinsic green nanostructures carrying C-16 side chain showed a maximum amount of incorporation through cell membranes, compared to C-18, and C-11 that showed a variable amount of incorporation in different developmental stages (Additional File 2 Figure S16A-B). Animals fed with C-18 self-assembled particles exhibit a maximum incorporation during larval stage as compared to other tested stages (Additional File 2 Figure S16A-B). Animals fed with C11 showed an overall low level of entry in all the stages of development.

Delivery of rhodamine B embedded nanoparticles C-12 showed an equal and maximal level of incorporation in all stages of development. The intensity was conspicuously greater than the nano-structure carrying C-14 chain (Additional File 2 Figure S16). Taken together, specific carbon chains and associated morphologies of nanostructures brought a potential difference in entry through gut cell walls. These results suggest the possibility that the physiology of gut cells in different stages of the life cycle might influence nanoparticles uptake.

For *in vivo *tracking, fluorescence dyes attached to nanoparticles suffer with multiple problems including photo-bleaching and ability to interrogate multiple targets etc. The aftermath effect of such limitations of fluorescence imaging in live objects was described earlier [21]. In all cases, during *in vivo *delivery there was no photobleaching of the nanomaterials through all stages of development providing a better advantage in tracking in live systems. But the fluorescence intensity was reduced conspicuously after extending the culture on an average of 18-25 days and nearly eliminated within 40-45 days allowing a total clearance of fluorescence from the live tissues. We further screened the efficacy of nano-particles inheritance through germ cells. The adult flies emerging from sole feeding of nanoparticle containing media were cultured in normal food media for another 7 days. The fertilized eggs from different batches of flies after nanoparticle feeding emits only trace amount of fluorescent as a background effect. Therefore, this ineffective route of gem cell based heritable transmission prevents nanomaterials contamination in the environment and their natural entry into the food chain via eco-consumers.

### Efficiency of organ specific delivery of PABA nanomaterials by side chain variation in *Drosophila*

**Figure 4:**
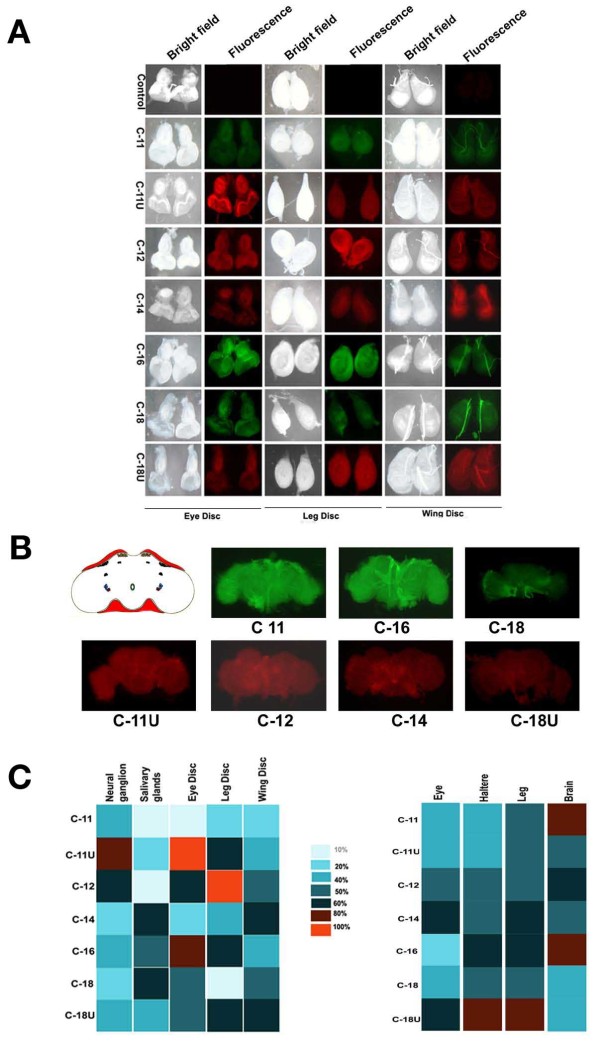
**Uptake and accumulation of orally deliverable 7 PABA nanomaterials**. (A) Uptake and accumulation of nanoparticles in eye, leg and wing imaginal discs, and (B) adult brain were shown. (C) Heat and intensity map representing larval discs specific uptake in complex adult tissues, eyes, halters, legs and brains of 7 PABA nanoparticles were presented. The different colour represents intensity of nanoparticles uptake (noted at the top). Each column represents mean values from six different experiments. The whitish blue refers to the lowest percentage of uptake (10%) and red refers to the highest accumulation (100%).

To categorize the intensity of fluorescent molecules as an absolute reflection on efficacy of nanoparticle delivery, different internal body parts of the larvae were dissected and visualized under fluorescence microscope. A wide range of variation in fluorescence intensity was observed in different larval body parts, for example mouth, brain, larval neural ganglia, salivary glands, alimentary canal and malpighian tubules etc (Figure 4; Additional File 2 Figure S16). A clear contrast was observed in the delivery of nanomaterials in the salivary glands. C-14 and C-18 containing nanostructures incorporated at a massive level in the glands but shows an intermediate level of incorporation in both neural tissues and organism itself (Figure 4). Surprisingly, we observed that malphigian tubules absorbed more nanoparticles that emit intrinsic fluorescence (Additional File 2 Figure S17). Therefore nanoparticles with different side chains showed a distinct distribution in various internal tissues in the larvae.

Nanoparticle entry showed a clear variation in rapidly dividing cells of mature larval imaginal discs (the precursor organs of adult wings, eyes and legs). PABA conjugated with C-16 side chain showed a higher intensity of uptake in all three discs tested. However the intensity of fluorescence is moderate in C-11U, C-12 and C-18 particles (Figure 4A, C). It suggests that the structure and surface texture of C-16 side chain is the most effective cargo for delivery in precursor and rapid dividing cells, though we can not rule out other unmet criteria in the tracking process (Figure 4A, C). As described above, the delivery of C-12 structure in all the stages of development is ideal compared to C-14 and nanomaterials with unsaturated side chains in C-11U and C-18U. Surprisingly a differential uptake of nanomaterials produced by C18 and C18U specially in leg discs that possess same number of carbon bonds interprets that length of the side chain is not an only criteria for nanoparticles based delivery in imaginal discs.

The conjugated side chains of PABA nanostructures were also screened for delivery to complex adult body parts derived from same sets of larval imaginal discs. Entry of nanomaterials was analysed in adult eyes, halters and legs. Incorporation in adult eyes is complicated and novel from other body parts. Two different fluorescent tags showed distinct uptake through eye ommatidia (Figure 4CAdditional File 2 Figure S18) raising the possibility that difference in fluorophore emission and structure make their entry visible and distinct in adult eyes. The intrinsic green showed a poor emission through ommatidia. Only a trace amount of green colour was visualized whereas rhodamine B showed a greater intensity with a maximum incorporation of C-16 in the eyes (Additional File 2 Figure S18). However, the incorporation pattern of nanomaterials conjugated with variable side chains in halters and legs is distinct from their distribution in eyes. Among all possible nanostructure, C-11U and C-18U were targeted orally at a maximal level to the legs while C-11, C-12 and C-16 in the halters showed an equal but greater amount of incorporation (Additional File 2 Figure 18), which suggests that the unsaturated carbon chains have advantage for selective entry in the accessory organs of *Drosophila*. Taken together, the delivery of nanoparticles associated with variable side chains in the culture cells and *in vivo *uptake by oral delivery in different body parts is different.

Furthermore distribution of numerous neurons and other cells make brain more compact and the delivery of therapeutic agents in the neuronal tissues is the most challenging task. In spite of complicated entry in brain, two nanoparticles, C-11 and C-16 containing particles show a considerable amount of entry when incorporation of other particles is nominal in the brain (Figure 4B). Truly, greater dissemination of nanostructures in adults, larvae and different body parts including brains suggests that physio-chemical properties including shapes, surface texture of the C-11 and C-16 particles are the best-fitted materials (Figure 4).

## Discussion

The key parameters of nanomaterials for easy and efficient delivery are shape, size and flexibility to enter and exit cell barrier. Our results clearly demonstrate that the properties of each acid side chain together with common PABA moiety influences size, shape and surface texture of nanomaterials that lead to differential uptake and specificity in live cell delivery. The physio-chemical modifications of organic nano carriers also affect cell internalization mechanisms in sub-cellular organelles as found by distinct accumulation pattern of each nanomaterials following same energy dependent endocytosis. *In vivo *screening also showed that only C 11 and C-16 produce compatible shape and size of nanomaterials that are best fitted for easy delivery of PABA nanomaterials. These results suggest that physical structure of nanomaterials and chemical properties of acid side chain required for self assemble procedure and size variation could be the initial step for cellular uptake.

In addition to cultured cells, tissue specific distribution specifically in adult eyes, imaginal discs, alimentary tracks and neuronal tissues was complex and needs more parameter to consider. Our data revealed that a complex interrelationship of PABA conjugates and cell physiological environment is important in live materials delivery. The internal tissue environment might provide additional barriers for nanomaterial entry as depicted by comparing variable accumulation of same nanomaterials in cross species; *Drosophila *and human cell lines. A similar difference was also noticed when C-11 or C16 accumulation was compared in multiple complex organ of *Drosophila*. However, nanomaterials compatible for oral delivery do not show any short-term toxicity, impaired growth of *Drosophila *larvae and adults [20,21]. We hypothesize that two distinct parameter nano-skeleton frame with conjugated acid chains and live cell physiology are best suited for cell uptake and delivery to internal organs after oral consumption.

Our results also differ from the hypothesis that nanoparticle uptake in live cells occurred through energy independent non-endocytotic pathway involving insertions and diffusion across the cell membrane. Sub cellular internalization of PABA nanomaterials predominantly takes place by energy dependent endocytosis. Earlier we have found that PABA nanomaterials can penetrate plasma membrane in the human cells and enter into cytoplasm. The variable amount of different nanomaterial accumulation by energy dependent endocytosis in same cell type ruled out the possibility that a single internalization mechanism, endocytosis is exclusively required for uptake. However, a marked reduction of different nanomaterials under endocytosis inhibitory conditions believed that such discrepancies are due to sharp differences in size and shape of the self assembled structures. In addition as organic nanomaterials suffer from uncontrolled aggregation to form micron sized particles after prolong storage; thereby ruling out the possibility of insertion, diffusion and penetration mechanisms [22]. PABA nanoparticles have a high tendency to associate with cell membrane (Figure 2, 3). Such accumulation might give rise to artefact in cellular uptake of micro-sized aggregates as found in artifactual intake of HIV TAT peptide at 4°C [23]. Therefore, cellular entry of PABA might depend on the size of the nanoparticles which is mainly guided by the acid side chain.

Finally, a systematic screening of PABA conjugated library provides sufficient evidences to support the following statements: 1) Two nanomaterials carrying C-11 and C16 acid side chains are best suited for optimal entry in cells and multiple organs. 2) In live tissues, an internal environment might be a useful barrier for improving nanoparticles delivery in multiple organs. 3) Cellular internalization or uptake mechanism of nanomaterials might unravel the clues for smooth entry in human cells and efficient delivery and 4) finally screening of PABA conjugates determine a functional relationship between energy dependent endocytosis and nanomaterial structure for each organ specific targeting.

## Conclusions

We have shown that C-11 and C-16 group of acid side chain forms tubular nanomaterials that are best fitted for oral delivery in complex multiorgans. The cellular uptake mechanism is energy dependent endocytosis. The detailed endocytosis pathways for nano PABA structure is operated thorough clathrin-coated pits rather than caveolae or lipid rafts. *In vivo *screening of PABA nanomaterials produced by different acid side chain select the compatible nano structure ideal for oral delivery and establish energy dependent entry mechanism is of fundamental importance that will facilitate future developments of PABA nanoparticle transporters for biological delivery application

## Methods

### Preparation of 4-Nitro-N-pyridine-2 yl-benzamide

As described earlier [12], the preparation of 4 nitro-N-pyridine-2 yl-benzamide is performed by mixing oxalyl chloride (5.68 mL, 65.8 mmol), catalytic DMF (dimethyl formamide) to a para nitro benzoic acid suspension (10 g, 59.8 mmol) in DCM (300 mL) at 0°C. The solution turned dark red by slowly adding tri-ethyl amine (24.48 mol, 179.4 mmol) at 0^o ^C. After 30 mins, 2-amino pyridine (6.198 g, 65.8 mmol) was mixed and stirred for overnight. The final precipitate was filtered and recrystalized in 70% acetic acid: water mixture to yield 10 g of 4-Nitro-N-pyridine-2 yl-benzamide (70%) (Additional File 1 Figure 2A).

### Preparation of 4-Amino-N-pyridine-2 yl-benzamide

For preparation of 4-Amino-N-pyridine-2 yl-benzamide, the suspension of 4-Nitro-N-pyridine-2 yl-benzamide (5.0 g, 20.5 mmol) in 75 mL of MeOH and 225 mL of DME (dimethoxy ethane) was slightly heated to form a clear solution initially as described elsewhere [12]. The 3.5 g of 10% Pd/C (palladium on activated carbon) was charged and hydrogenation was carried out as preset condition. The white solid compound, 4-Amino-N-pyridine-2 yl-benzamide 4 (95%) (Additional File 1 Figure 2B) was formed, which is further used for next reaction.

### General procedure for preparation of compounds (1)

To a mixture of 0.5 g (2.34 mmol) of 4-Amino-N-pyridine-2 yl-benzamide 4 and 2 ml pyridine in dry THF (15 ml) was added to respective acid chlorides (2 equivalent) following the same protocol as described earlier [12]

### Cell Culture

Two regular human cell lines, Human HEK-293 and HeLa cells were selected to grow in Dulbecco's Modified Eagle's Medium (Sigma Chemical, USA) supplemented with 10% fetal bovine serum and common antibiotics (penicillin, kanamycin, and streptomycin) at 1× concentration. Cells were routinely maintained in a standard humidified atmosphere of 5% CO_2 _at 37°C. and further sub-cultured in every three days interval. The cells were seeded in a concentration of 1 × 10^6 ^per ml, nearly 24 hours prior to treatment in 6 well plates and cover slips for further studies in MTT assay by flow cytometry and Confocal microscopy etc The seeding media was removed completely after 24 hours, cells adhered to the plate surface were washed with PBS gently and further fresh media was added. The cultures were incubated with Dimethyl Sulfoxide (0.01% DMSO) containing different conjugated PABA nanoparticles at optimized concentrations (60 ug/ml) and harvested after 24, 48 and 72 hrs [12]. The cultures only incubated in same DMSO (0.01%) buffer without any nanomaterials serves as internal control.

### FACS and MTT Assay

The cell proliferation was determined by colorimetric assay using 3-(4,5 dimethylthiazol-2yl)-2,5 diphenyltetrazolium bromide (MTT). The assay is based on reductive capacity to metabolize the tetrazolium salt to blue colored formazone. The cultured cells seeded on 96 well microplates nearly 6000 cells/well were incubated for 48 hrs with various concentrations of nanotubes containing fresh media. The medium was changed once with fresh culture medium in 24 hr interval. MTT assay was performed after 1, 2 or 3 days as described earlier [24]. Briefly, cells were incubated with 0.5 mg/ml of MTT (Sigma) for 4 hr in a CO2 incubator at 37°C. After the removal of the solution, the purple precipitates were dissolved in DMSO for 20 min at room temperature and the resultant solution was transferred to new 96-well plates. The absorbance was measured at 570 nm using a Lab systems Multiscan RC enzyme-linked immunosorbent assay (ELISA) reader. Each experiment was performed in triplicate, and the means were determined for each time point.

For the FACS assay, cells were pelleted and fixed with ice cold 80% methanol overnight. They were stained with 20 ug/ml of Propidium Iodide (PI) and analysis was done on the MoFlow-Dako Cytomation (Dako, Denmark).

### Confocal Microscopy

For microscopic studies, the cells cultured on the coverslips were washed with PBS and fixed with 4% para-formaldehyde for 20 mins followed by a PBS wash. The fixed cells containing coverslips were then mounted on microscopic slides with 80% glycerol. The intrinsic fluorescence and rhodamine-B was excited at the 488 nm and 543 nm laser respectively. Confocal images were viewed on Olympus FV1000 laser microscope and captured and processed by Adobe photoshop software (version CS2).

### Measurement of fluorescent intensity

As noted earlier, the measurement of fluorescent intensity is proportional to the incorporation of nanomaterials accumulation in an individual cells or different organs of the whole organism [25]. The intrinsic green and rhodamin B have excitation at 488 and 543 nm. The relative amount of nanoparticles/specified area was calculated by determining the gray scale values using Metamorph version 4.6 soft ware. Gray scale, which is defined as brightness of pixel in a digital image is an eight-bit digital signal with 256 possible values ranging from 255 (white) to 0 (black). The average grey values are equivalent to the ratio of total gray scale values per number of pixels. Measuring the mean gray scale values, the total (sum of) gray scale values of entire designated area were initially calculated and divided by the total pixel number of the entire area.

## Abbreviations

PABA-Para aminobenzoic acid, THF- Tetrahydrofuran, HEK- Human embryonic Kidney, FACS-Fluorescence Activated Cell Sorting, MTT-3-(4,5-Dimethylthiazol-2-yl)-2,5-diphenyltetrazolium bromide, ELISA-Enzyme-linked immunosorbent assay, PBS-phosphate buffer saline, U- unsaturated, C-carbon bond

## Competing interests

The authors declare that they have no competing interests

## Authors' contributions

JY participated in designing of nanomaterials and review the manuscript. PP and LNR synthesized and characterized the nanoparticles physically. LNR performed sub-cellular internalization experiments. IB and PL conducted all microscopy and *in vivo *experiment in *Drosophila*. BJ and DKM participated in evaluation of chemical properties of nanomaterials and supplied information for writing final manuscript. MB and UB design all biological experiments and wrote the final manuscript. All authors read and approved the final manuscripts.

## Supplementary Material

Additional file 1 Synthetic scheme of nanoparicle, Chemical Structure of 4-Nitro-N-pyridine-2 yl-benzamide and 4-Amino-N-pyridine-2 yl-benzamide Click here for file

Additional file 2 **Supporting online information http://www.Jnnanobiotecnology.com/imedia/1579050806614326/supp1.doc**. Click here for file
