# Supplementary material for: Sub-cellular internalization and organ specific oral elivery of PABA nanoparticles by side chain variation
Source: J Nanobiotechnology. 2011 Mar 28;9:10. doi: 10.1186/1477-3155-9-10 (PMC3076233; doi:10.1186/1477-3155-9-10)

Additional File 1

Subcellular internalization and organ targeted oral delivery of PABA nanoparticles by side chain variation

Jhillu S. Yadav*[1,], Pragna P. Das[1], T. Lakshminarayan Reddy[1], Indira Bag[1], Priyadarshini M. Lavanya[2], Bulusu Jagannadh[1], Debendra K. Mohapatra[1], Manika Pal Bhadra[1] and Utpal Bhadra*[2]

Figure.1 Synthetic scheme of Nanoparticles.

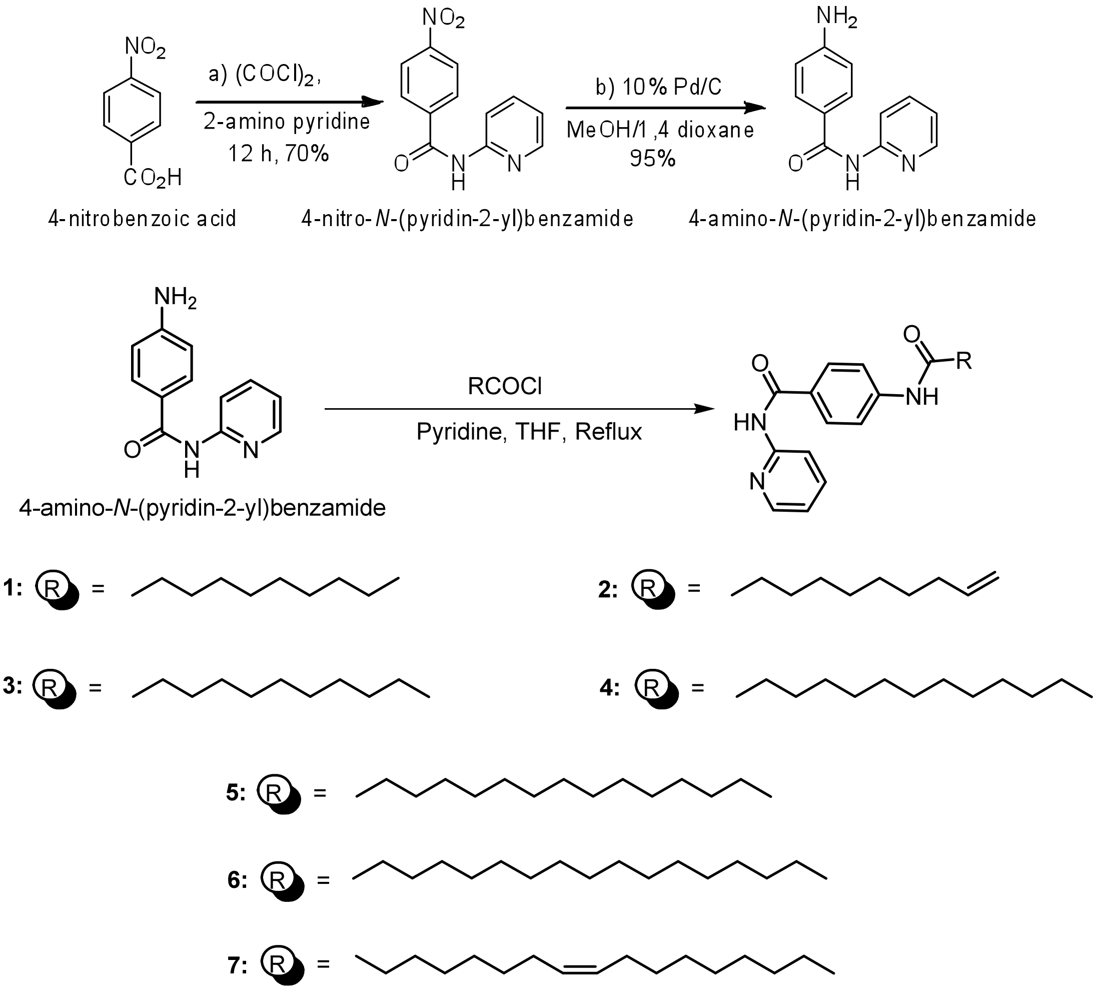

Figure 2A: 4-Amino-N-pyridine-2yl-benzamide

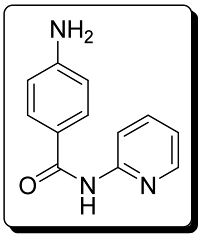

Figure 2B: 4-Nitro-N-pyridine-2yl-benzamide

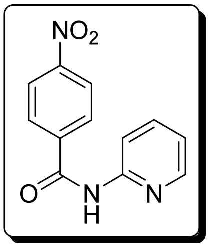

Supplement: Additional file 1 — Synthetic scheme of nanoparicle, Chemical Structure of 4-Nitro-N-pyridine-2 yl-benzamide and 4-Amino-N-pyridine-2 yl-benzamide [file 1477-3155-9-10-S1.PDF]
