# Supplementary material for: Sub-cellular internalization and organ specific oral elivery of PABA nanoparticles by side chain variation
Source: J Nanobiotechnology. 2011 Mar 28;9:10. doi: 10.1186/1477-3155-9-10 (PMC3076233; doi:10.1186/1477-3155-9-10)
Supplement: Additional file 2 — Supporting online information http://www.Jnnanobiotecnology.com/imedia/1579050806614326/supp1.doc. [file 1477-3155-9-10-S2.PDF]

Additional File 2

Subcellular internalization and organ targeted oral delivery of PABA nanoparticles by side chain variation

Jhillu S. Yadav*[1,], Pragna P. Das[1], T. Lakshminarayan Reddy[1], Indira Bag[1], Priyadarshini M. Lavanya[2], Bulusu Jagannadh[1], Debendra K. Mohapatra[1], Manika Pal Bhadra[1] and Utpal Bhadra*[2]

Characterization of the compounds

C-11U:

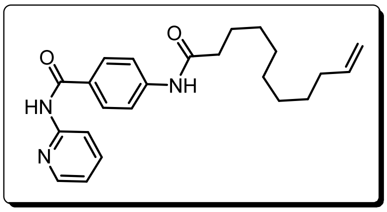

Melting point : 118-122 °C; ^{1}H NMR ($CDCl_3$, 300 MHz): δ 9.41 (br s, 1H), 8.44 (d, *J* = 8.3 Hz, 1H), 8.25 (d, *J* = 4.5 Hz, 1H), 8.09 (s, 1H), 7.92 (d, *J* = 8.3 Hz, 2H), 7.80 (t, *J* = 6.2 Hz, 1H), 7.59 (d, *J* = 8.3 Hz, 2H), 7.10 (t, *J* = 6.2 Hz, 1H), 5.69 (m, 1H), 5.02-4.88 (m, 2H), 2.39 (t, *J* = 7.4 Hz, 2H), 2.08-1.98 (m, 2H), 1.76-1.66 (m, 2H), 1.40-1.23 (m, 10H); ^{13}C NMR ($CDCl_3$, 300 MHz): δ 172.3, 165.3, 151.4, 146.4, 142.2, 139.0, 128.4, 128.3, 119.6, 119.1, 114.6, 114.0, 37.5, 33.6, 29.2, 28.9, 28.7, 25.4; ESI-HRMS calcd for $C_{23}H_{29}N_3O_2$: *m/z* [M+H]+ 380.2338, found 380.2340;

C-11:

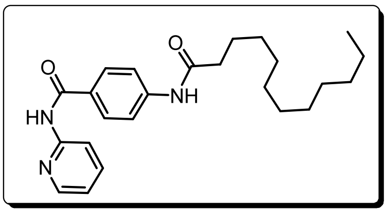

Melting point : 125-126 °C ^{1}H NMR ($CDCl_3$, 300 MHz): 8.56 (br s, 1H), 8.37 (d, *J* = 8.1 Hz, 1H), 8.30 (br s, 1H), 7.90 (d, *J* = 8.1 Hz, 2H), 7.75 (t, *J* = 7.4 Hz, 1H), 7.67 (d,

J = 7.9 Hz, 2H), 7.39 (s, 1H), 7.07 (t, *J* = 5.7 Hz, 1H), 2.39 (t, *J* = 7.2 Hz, 2H), 1.80-1.68 (m, 2H), 1.40-1.21 (m, 14 H), 0.88 (t, *J* = 6.2 Hz, 3H); ^{13}C NMR ($CDCl_3$+DMSO, 75 MHz) δ: 172.5, 165.1, 151.5, 147.1, 142.4, 138.3, 128.1, 119.4, 118.9, 114.1, 37.2, 31.5, 29.3, 29.2, 29.1, 29.0, 28.9, 25.2, 22.3, 13.8; ESI-HRMS calcd for $C_{23}H_{31}N_3O_2$: *m/z* [M+Na]+ 404.2313, found 404.2334;

C-14:

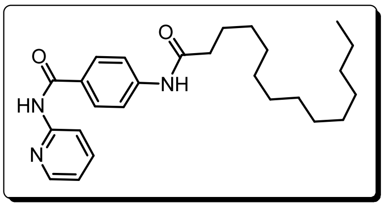

Melting point : 130-132 °C ^{1}H NMR ($CDCl_3$+DMSO, 300 MHz) δ: 10.24 (br s, 1H), 9.53 (s, 1H), 8.53 (d, *J* = 8.5 Hz, 1H), 833 (d, *J* = 8.5 Hz, 1H), 7.80 (d, *J* = 8.7 Hz, 2H), 7.19 (t, *J* = 6.2 Hz, 1H), 2.40 (t, J = 7.4 Hz, 2H), 1.76-1.66 (m, 2H), 1.44-1.20 (m, 22H), 0.88 (t, *J* = 6.8 Hz, 3H); ^{13}C NMR ($CDCl_3$+DMSO, 75 MHz): δ 172.3, 165.3, 151.2, 145.7, 142.7, 139.3, 128.3, 119.3, 118.9, 114.6, 37.2, 31.6, 29.3, 29.2, 29.1, 29.0, 25.2, 22.3, 13.8; ESI-HRMS calcd for $C_{26}H_{37}N_3O_2$: *m/z* $[M+H]^+$ 424.2964, found 424.2949;

C-16:

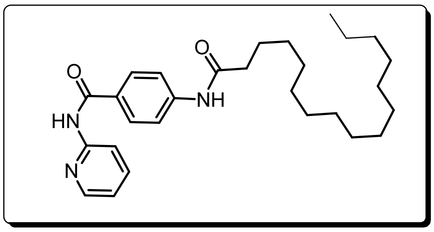

Melting point : 139-140 °C; ^{1}H NMR ($CDCl_3$+DMSO, 300 MHz) δ: 10.36 (br s, 1H), 9.22 (s, 1H), 8.65 (br s, 1H), 8.09 (d, *J* = 8.5 Hz, 2H), 7.99 (t, *J* = 7.2 Hz, 1H), 7.81 (d, *J* = 8.3 Hz, 2H), 7.36 (s, 1H), 7.25 (bs, 1H), 2.41 (t, *J* = 7.7 Hz, 2H), 1.78-1.67 (m, 2H), 1.40-1.20 (m, 26H), 0.89 (t, *J* = 7.0 Hz, 3H); ^{13}C NMR ($CDCl_3$+DMSO, 75 MHz): δ 172.2, 165.3, 150.9, 144.8, 142.8, 139.7, 128.3, 127.2, 119.2, 118.7, 114.7, 36.9, 31.4, 29.2, 29.0, 28.9, 28.8, 25.1, 22.2, 13.7; ESI-HRMS calcd for $C_{28}H_{41}N_3O_2$: *m/z* [M+H]+ 452.3277, found 452.3298;

C-18U:

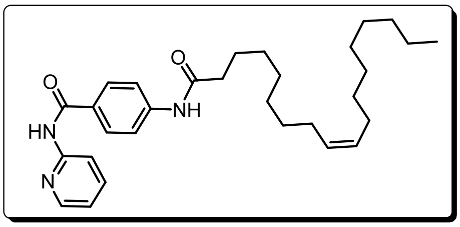

Melting point : 134-135 °C; ^{1}H NMR ($CDCl_3$+DMSO, 300 MHz) δ: 9.40 (br s, 1H), 8.41 (d, *J* = 8.5 Hz, 1H), 8.24-8.20 (m, 2H), 7.88 (d, *J* = 8.7 Hz, 2H), 7.76 (m, 1H), 7.64 (d, *J* = 8.7 Hz, 2H), 7.06 (t, *J* = 7.2 Hz, 1H), 5.35-5.26 (m, 2H), 2.37 (t, *J* = 7.5 Hz, 2H), 2.04-1.94 (m, 4H), 1.74-1.64 (m, 2H), 1.36-1.21 (m, 20H), 0.87 (t, *J* = 7.0 Hz, 3H); ^{13}C NMR ($CDCl_3$+DMSO, 75 MHz) δ: 172.1, 165.3, 151.4, 146.9, 142.0, 138.9, 129.9, 129.6, 128.8, 128.4, 119.8, 119.2, 114.5, 37.6, 31.8, 29.7, 29.4, 29.2, 27.1, 25.4, 22.6, 14.0; ESI-HRMS calcd for $C_{30}H_{43}N_3O_2$: *m/z* [M+H]+ 478.3434, found 478.3425;

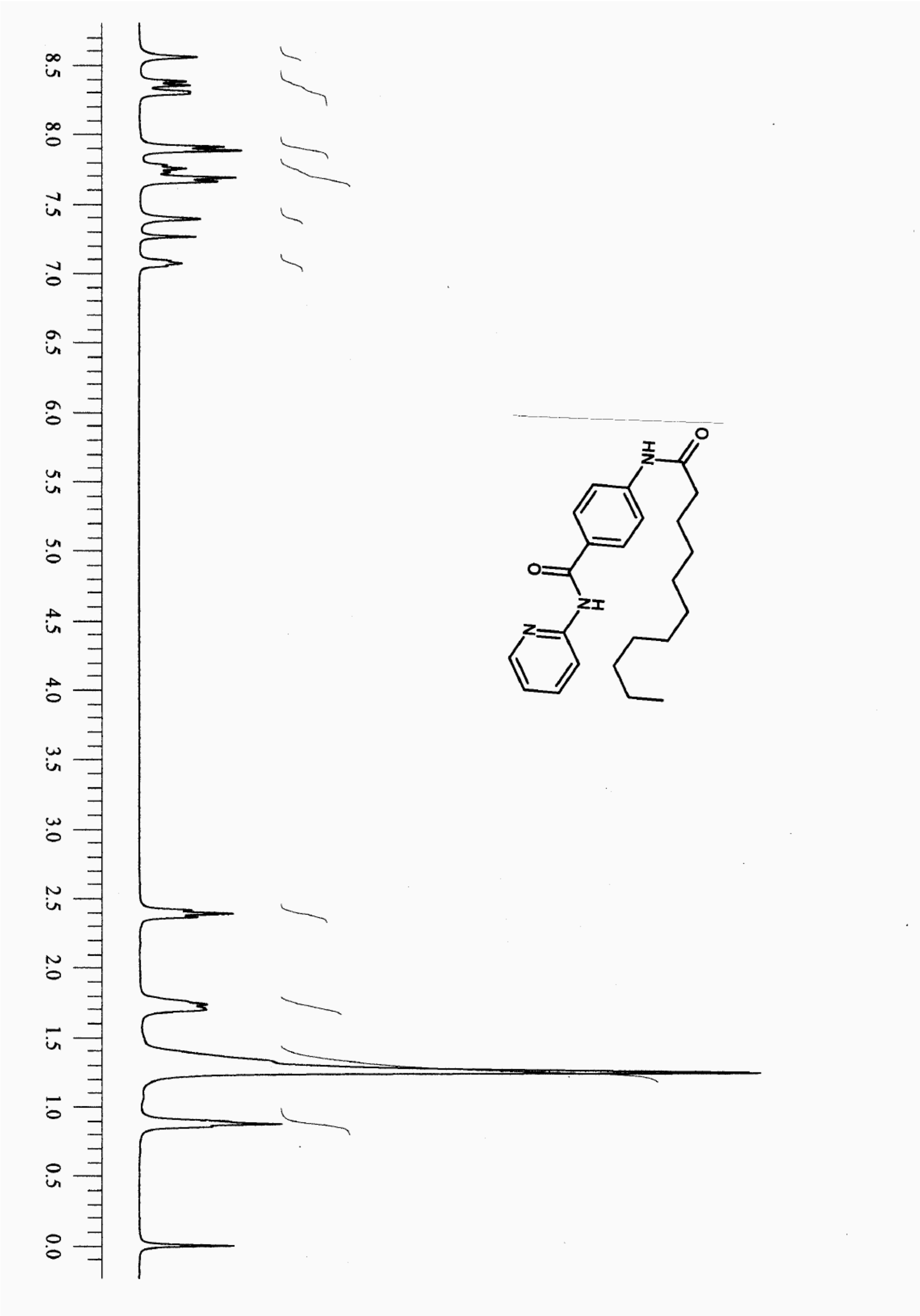

Figure S1. ^{1}H NMR Spectrum of Compound **1 (C-11)**

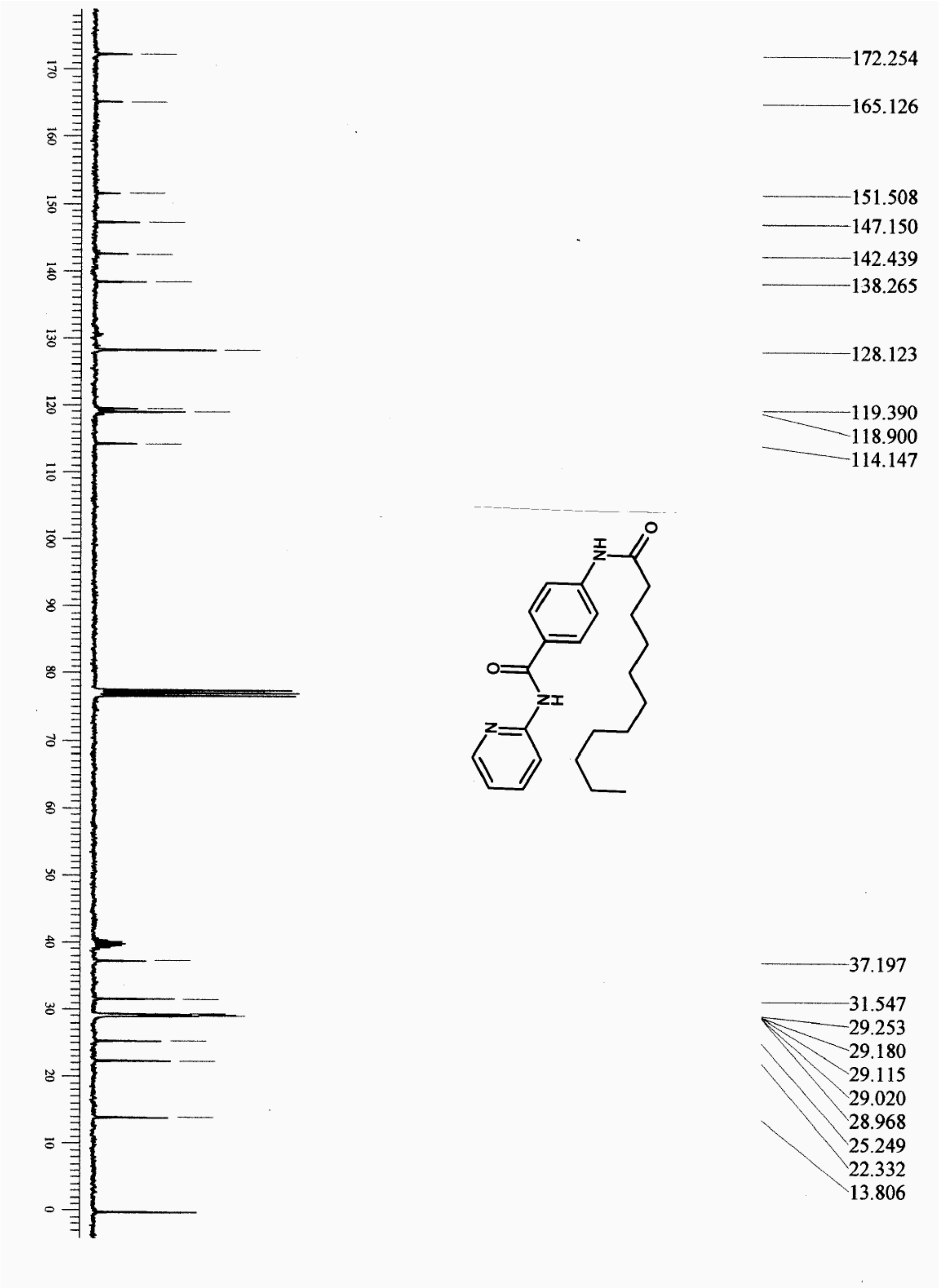

Figure S2. ^{13}C NMR Spectrum of Compound 1 (C-11)

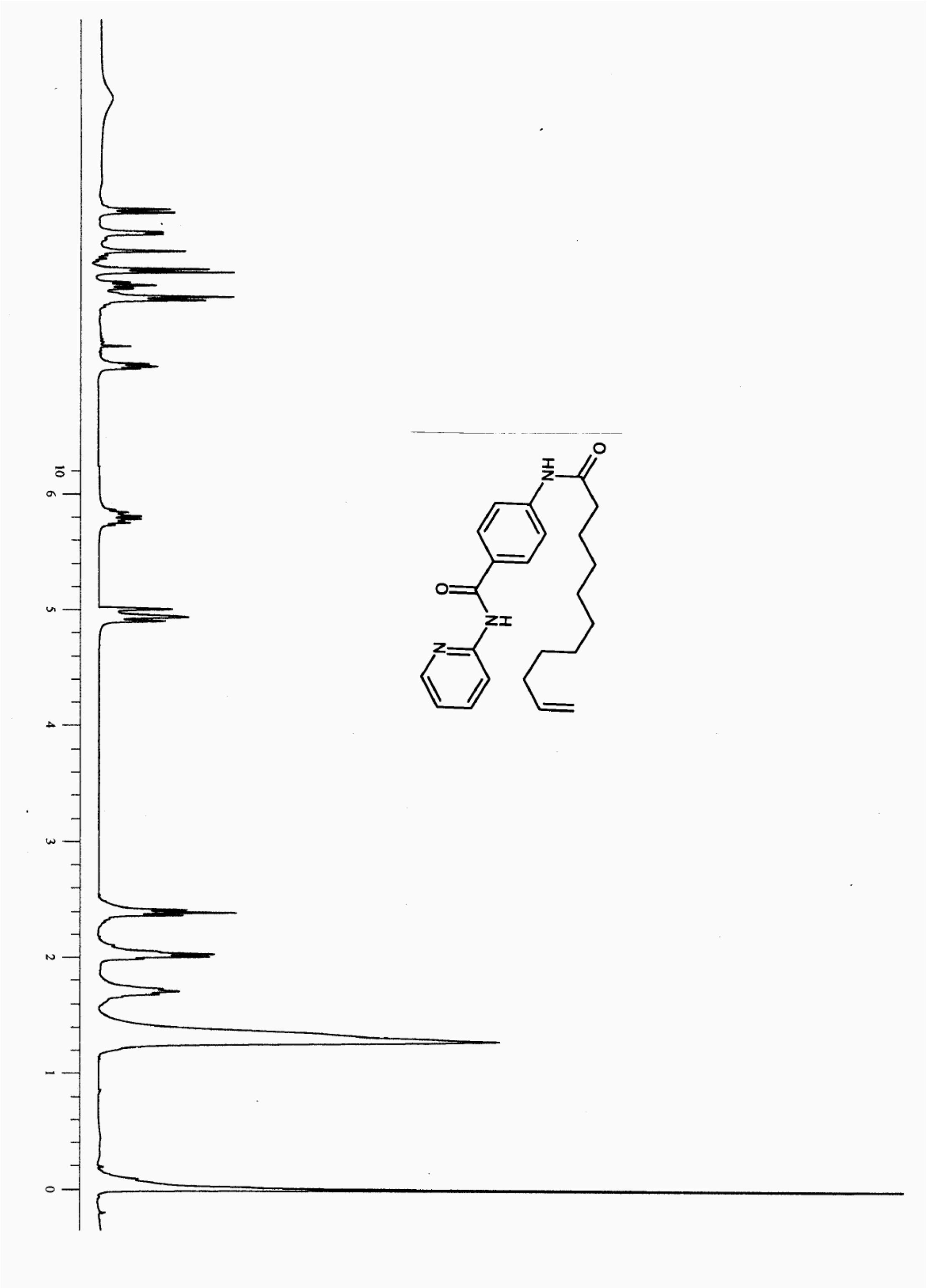

Figure S3. ^{1}H NMR Spectrum of Compound 2 (C-11U)

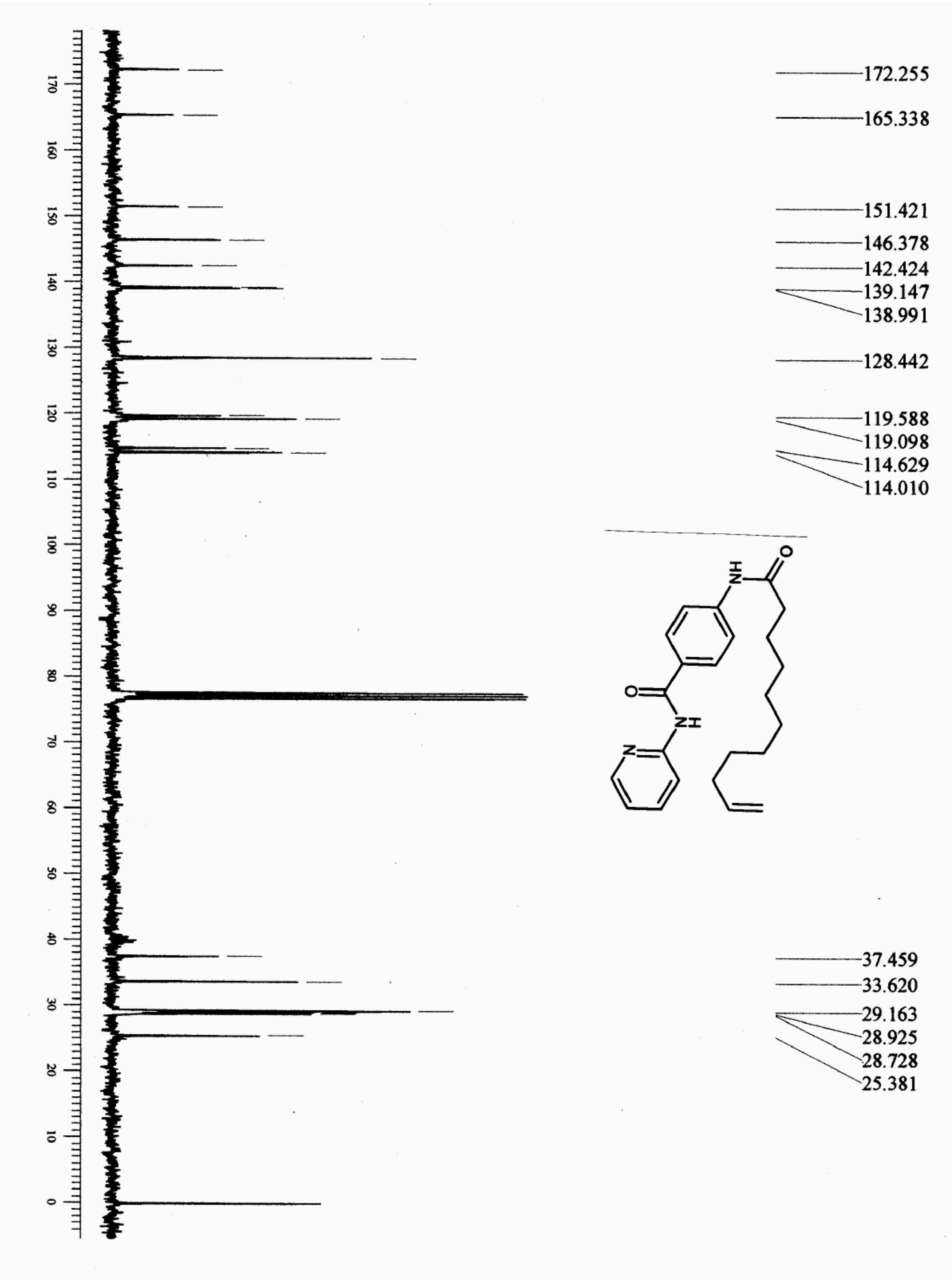

Figure S4. ^{13}C NMR Spectrum of Compound 2 (C-11U)

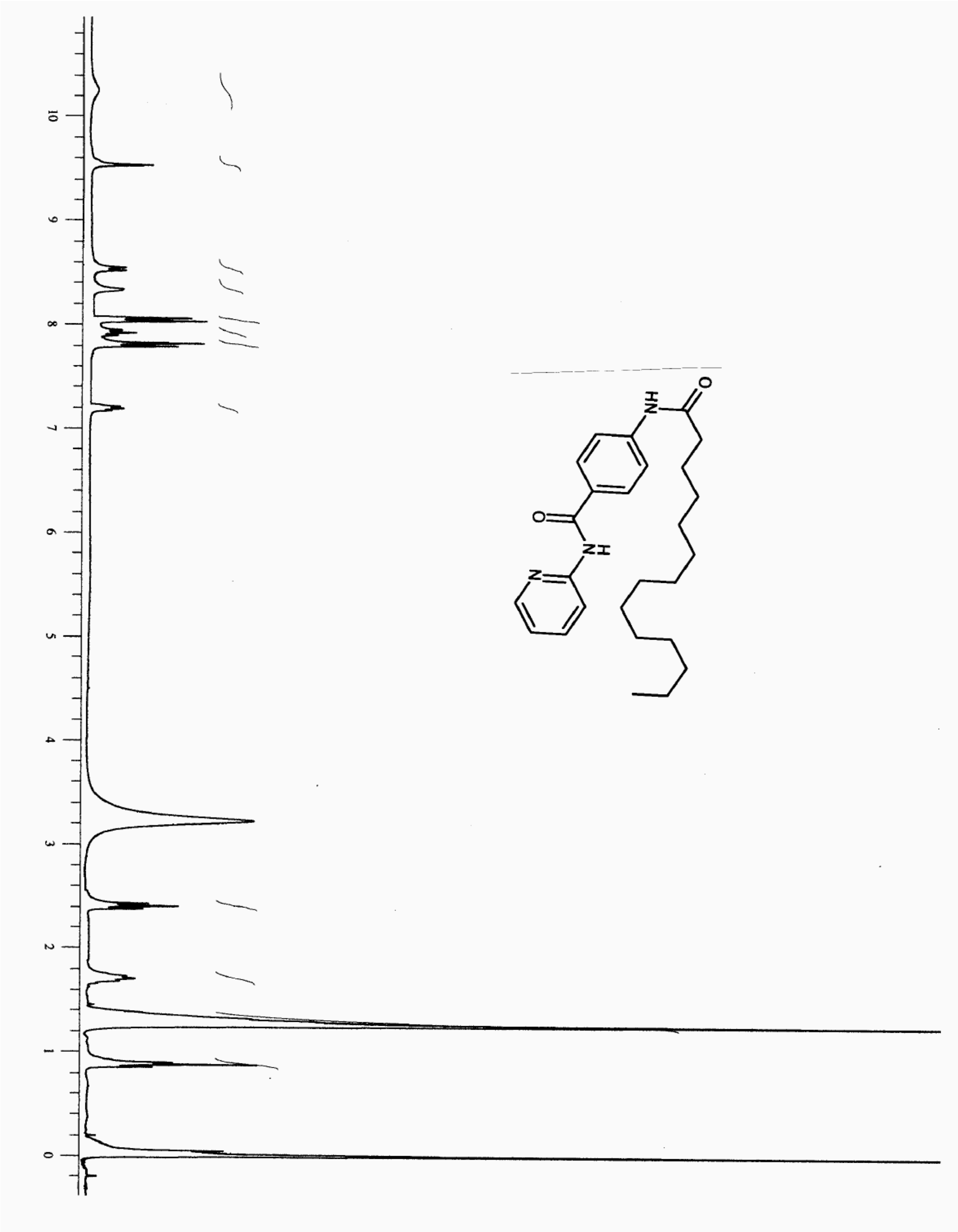

Figure S5. ^{1}H NMR Spectrum of Compound 4 (C-14)

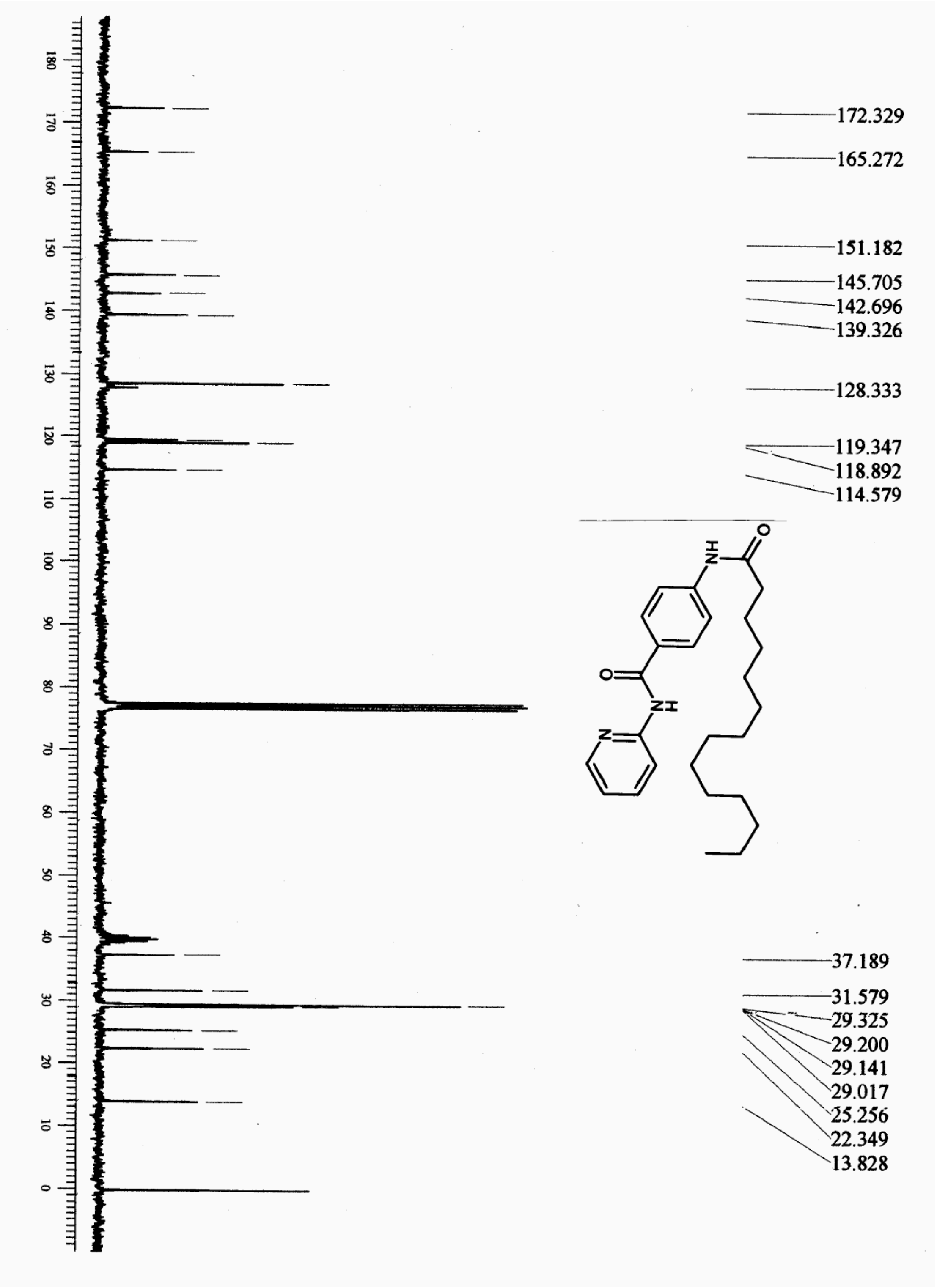

Figure S6. ^{13}C NMR Spectrum of Compound 4 (C-14)

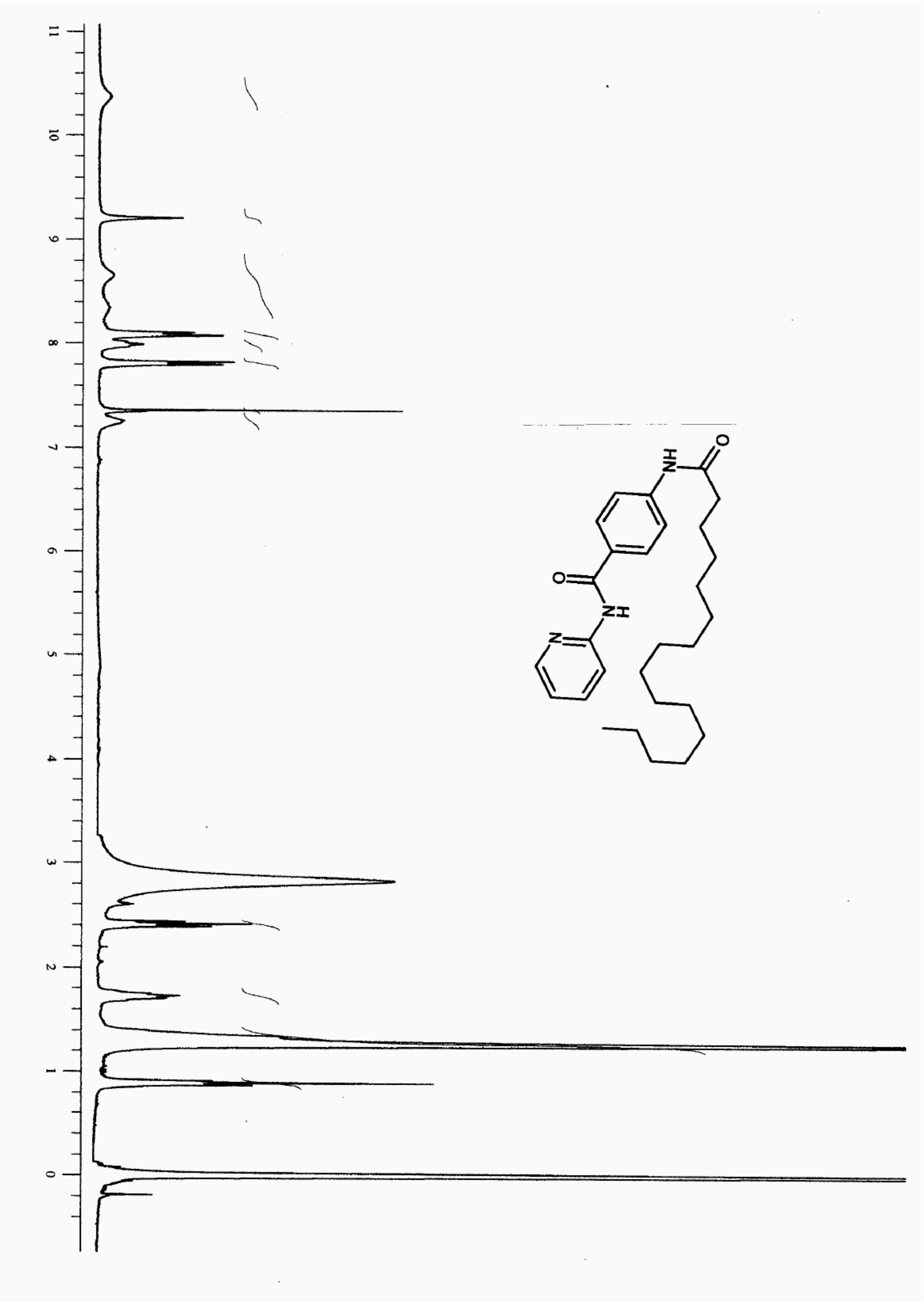

Figure S7. ^{1}H NMR Spectrum of Compound 5 (C-16)

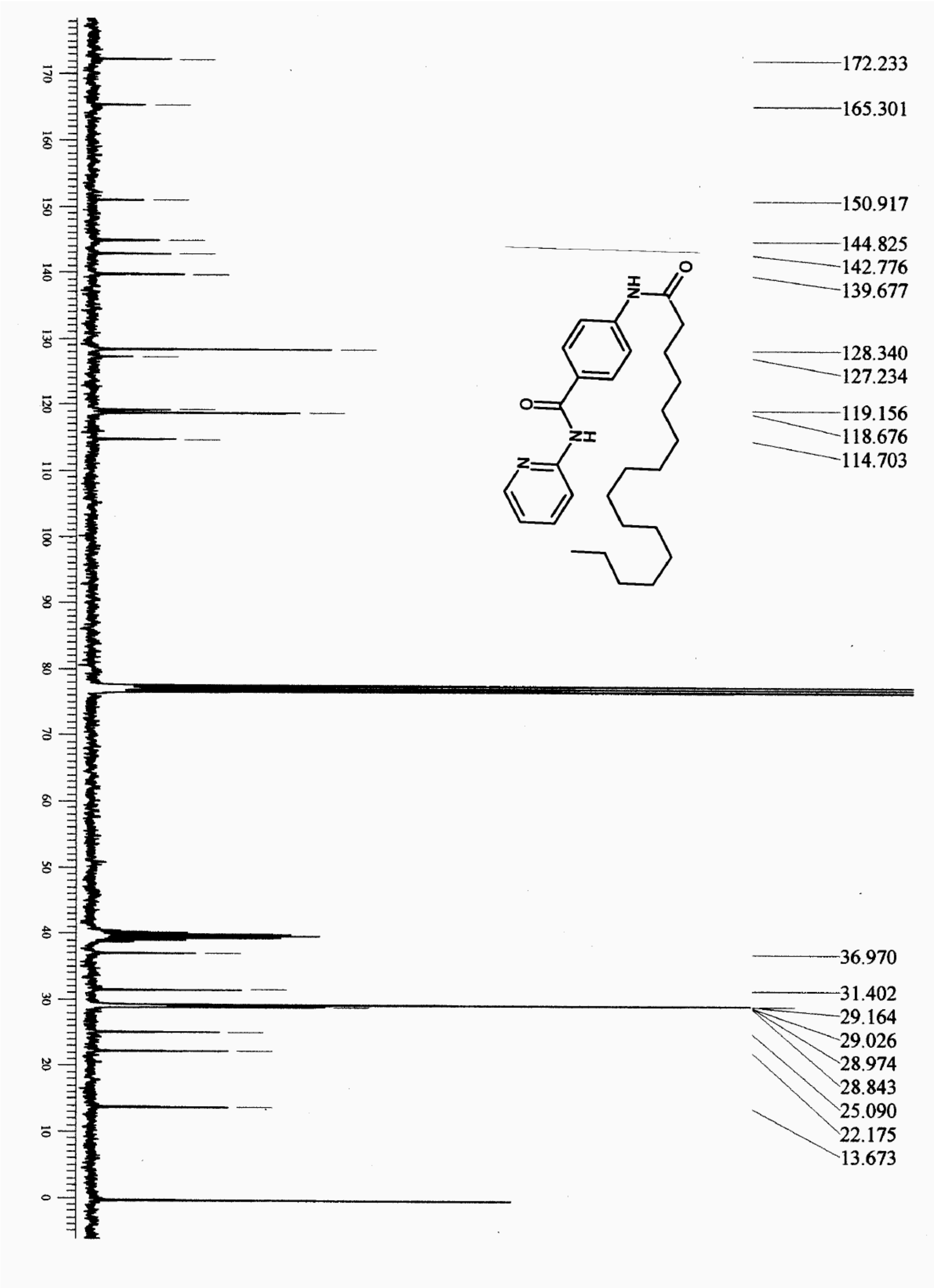

Figure S8. ^{13}C NMR Spectrum of Compound 5 (C-16)

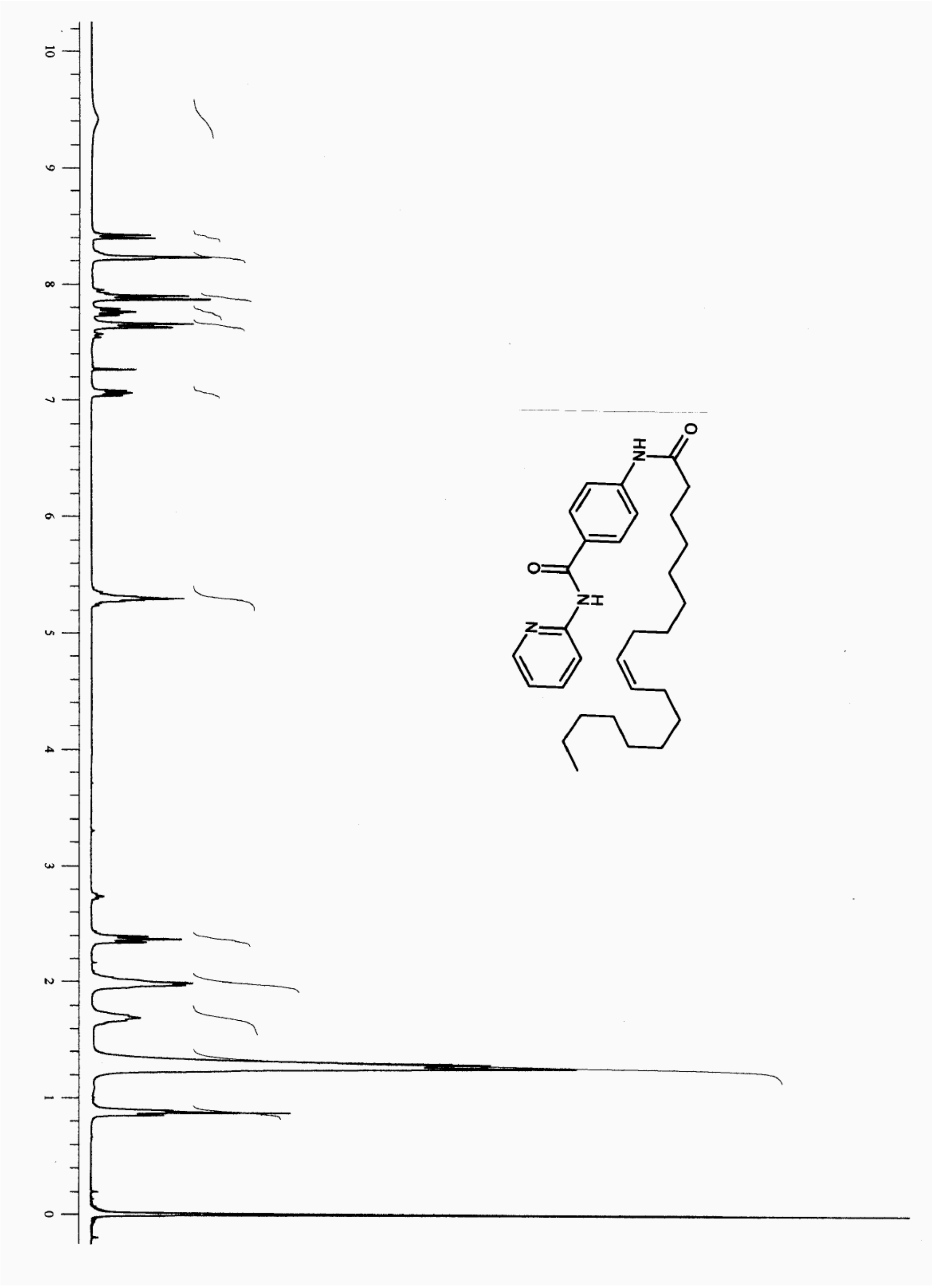

Figure S9. ^{1}H NMR Spectrum of Compound 7 (C-18U)

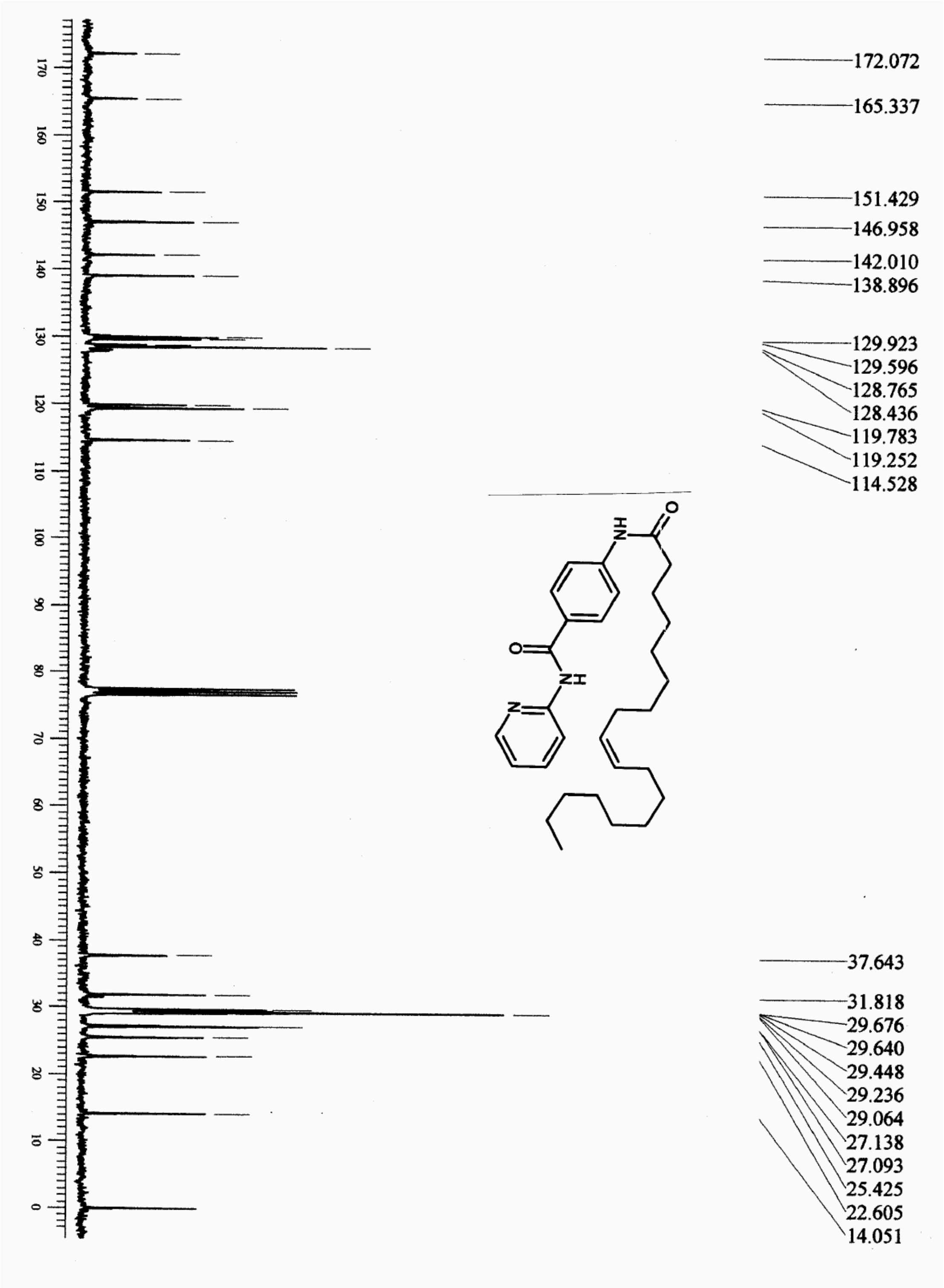

Figure S10. ^{13}C NMR Spectrum of Compound 7 (C-18U)

C-11

		Diam. (nm)	% Intensity	Width (nm)
	Peak 1:	99.13	100.0	7.187
PdI: 0.884	Peak 2:	0.000	0.0	0.000
Intercept: 0.937	Peak 3:	0.000	0.0	0.000

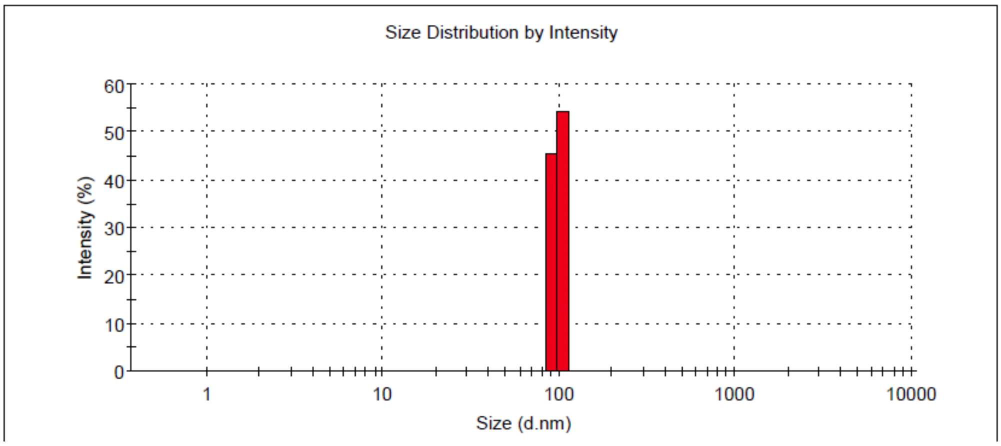

C-11U

		Diam. (nm)	% Intensity	Width (nm)
	Peak 1:	91.28	100.0	0.000
PdI: 1.000	Peak 2:	0.000	0.0	0.000
Intercept: 0.967	Peak 3:	0.000	0.0	0.000

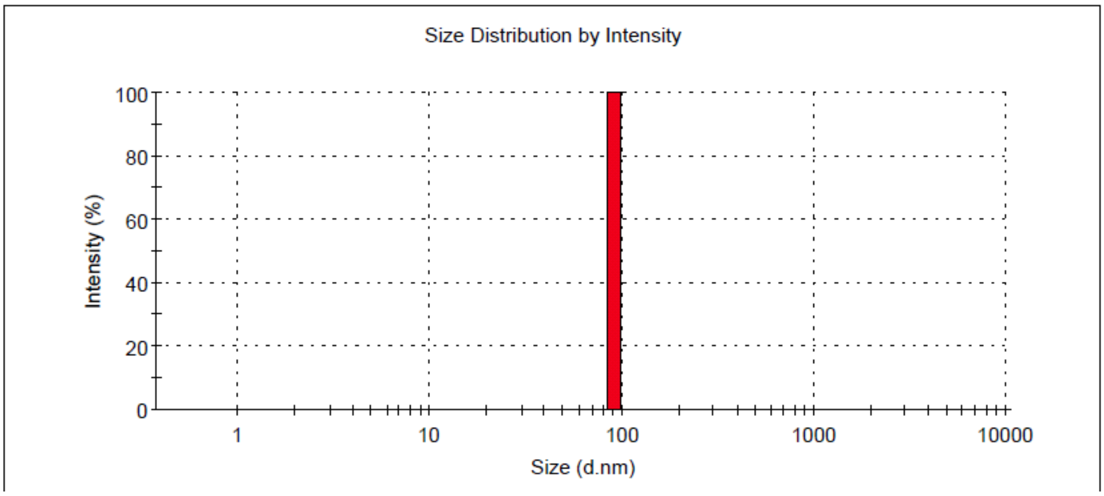

C-14

		Diam. (nm)	% Intensity	Width (nm)
	**Peak 1:**	410.5	100.0	35.18
**PdI: 0.607**	**Peak 2:**	0.000	0.0	0.000
**Intercept:** 0.730	**Peak 3:**	0.000	0.0	0.000

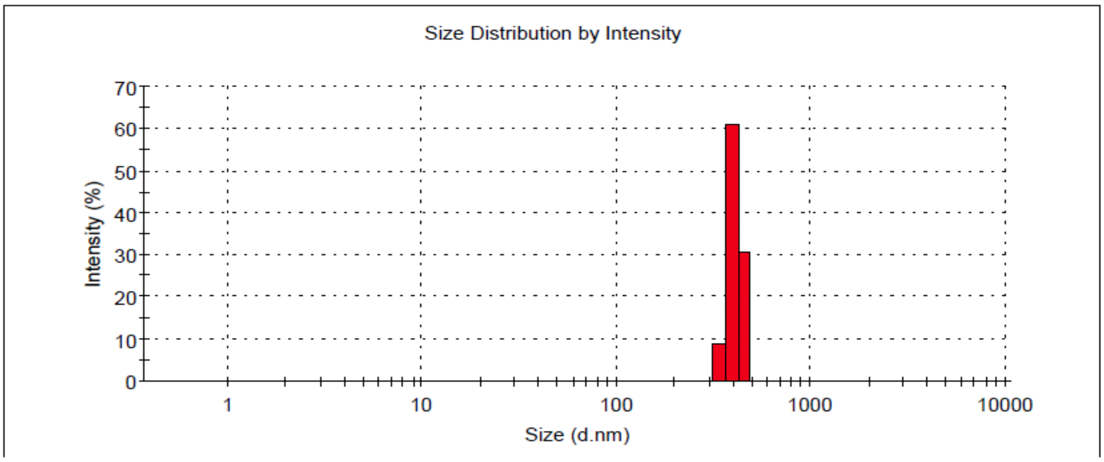

C-16

		Diam. (nm)	% Intensity	Width (nm)
	**Peak 1:**	348.4	100.0	50.07
**PdI: 0.639**	**Peak 2:**	0.000	0.0	0.000
**Intercept:** 0.443	**Peak 3:**	0.000	0.0	0.000

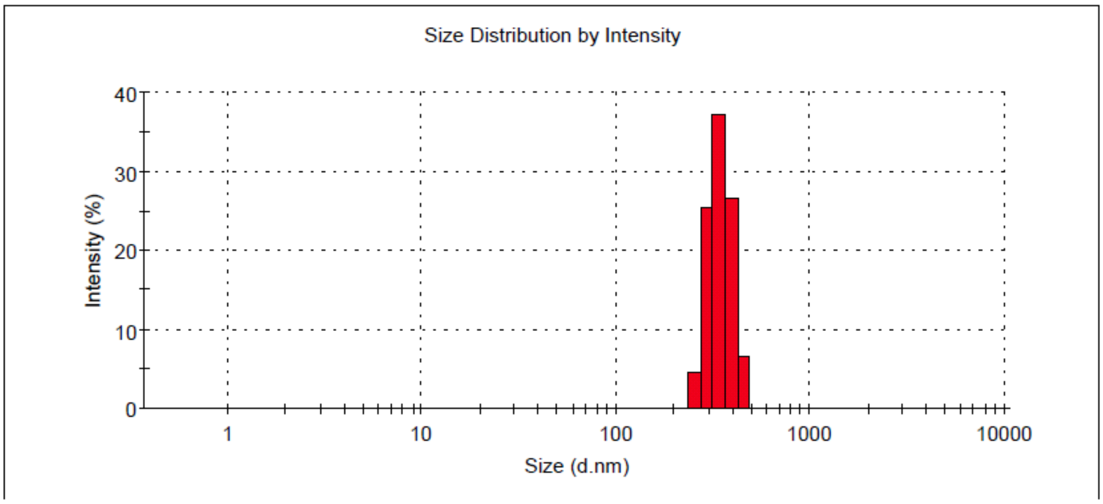

C-18U

		Diam. (nm)	% Intensity	Width (nm)
	Peak 1:	134.5	100.0	9.365
**PdI: 1.000**	Peak 2:	0.000	0.0	0.000
Intercept: 1.28	Peak 3:	0.000	0.0	0.000

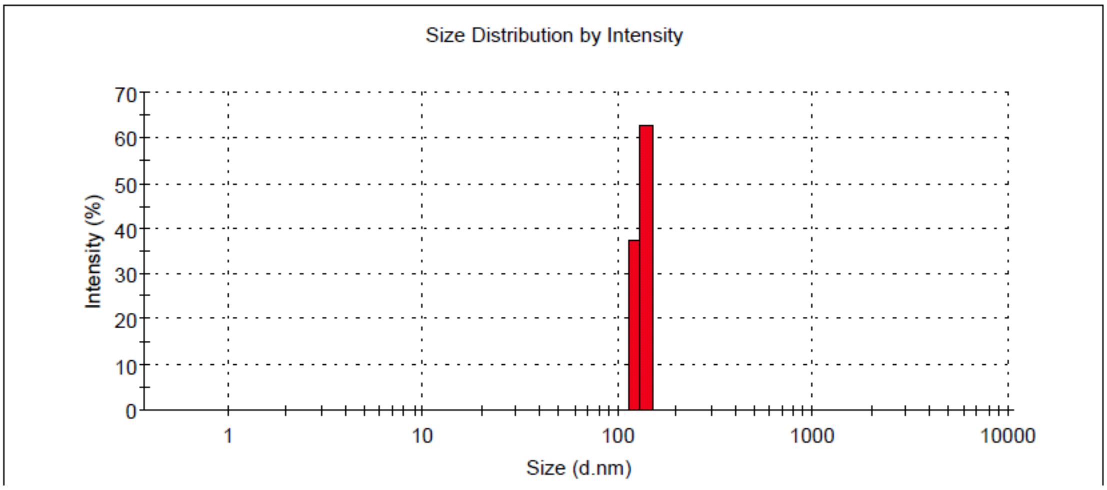

Figure S11. The average particle size in nanometers and poly dispersity index (Pdi) of the developing formulations of different PABA nanomaterials were measured by photon correlation spectroscopy using Zetasizer Nano ZS (Malvern Instruments, UK). The prepared formulations were diluted with de-ionized water or the aqueous phase of the formulation to get optimum kilo counts per second (Kcps) of 50 to 200 for measurements.

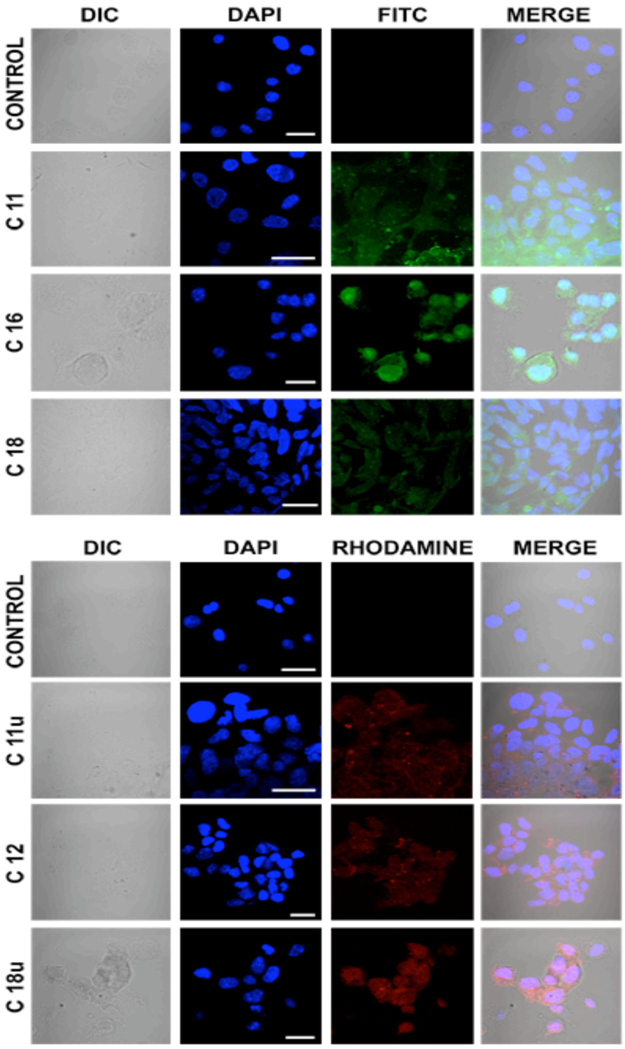

Figure S12. Biocompatibility of nanoparticles in nonneoplastic (HEK-293) Human Embryonic Kidney cells. Specificity of cellular uptake of different nanoparticles in HEK-293 cells was shown. The cells were incubated in 0.1% DMSO (control) and 60mg/ml of each nanoparticle containing culture media separately for 12 hrs prior to process. The cells were counterstained with DAPI. The DIC images and merge figures were shown in the left and right sides of the panel. Scale 40μm.

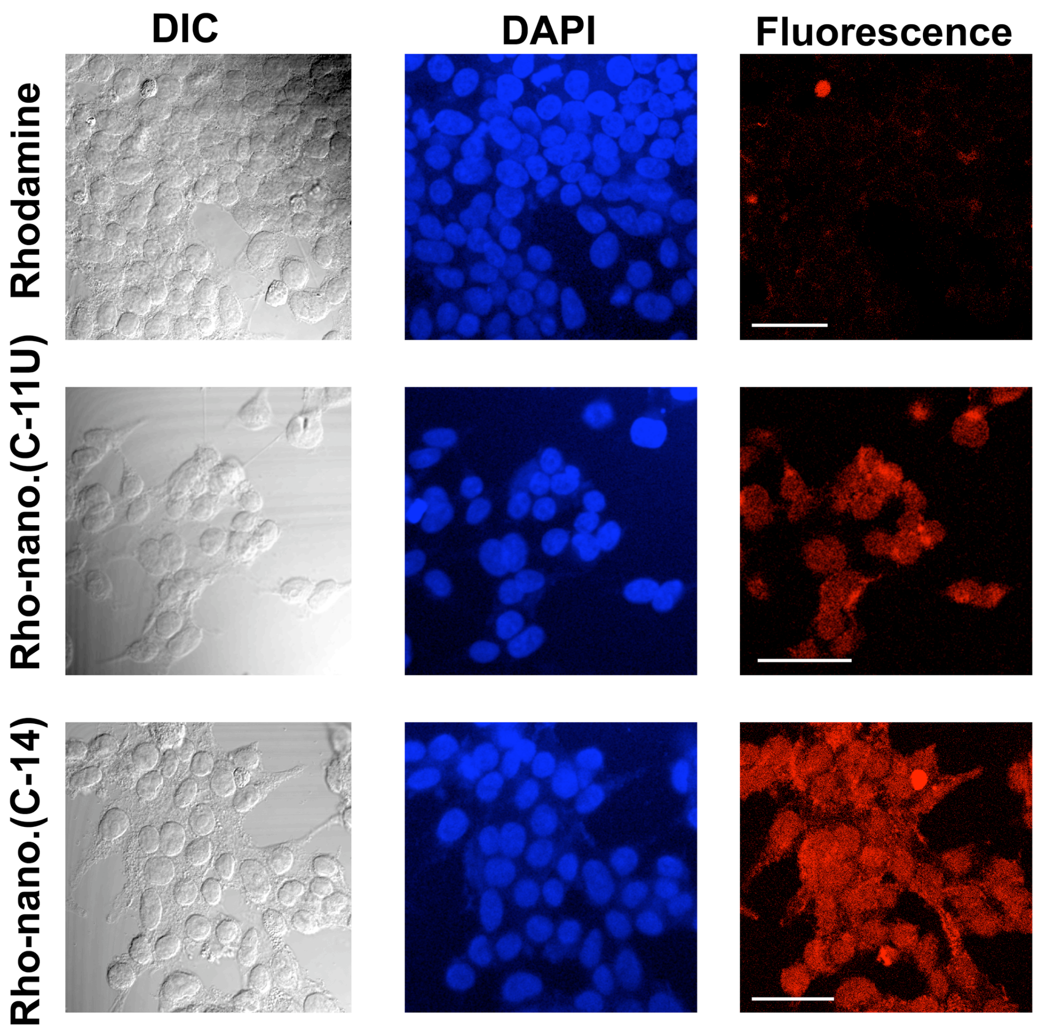

Figure S13 Uptake of raw rhodamine were compared to rhodamine containing nanoparticles uptake in HEK-293 cultured cell lines**.** Three separate sets of HEK-293 cells were cultures with raw rhodamine, C-11U and C-14 at 60 µg/ml concentration. The cells were fixed and processed and viewed in confocal microscope. Scale -50 µm.

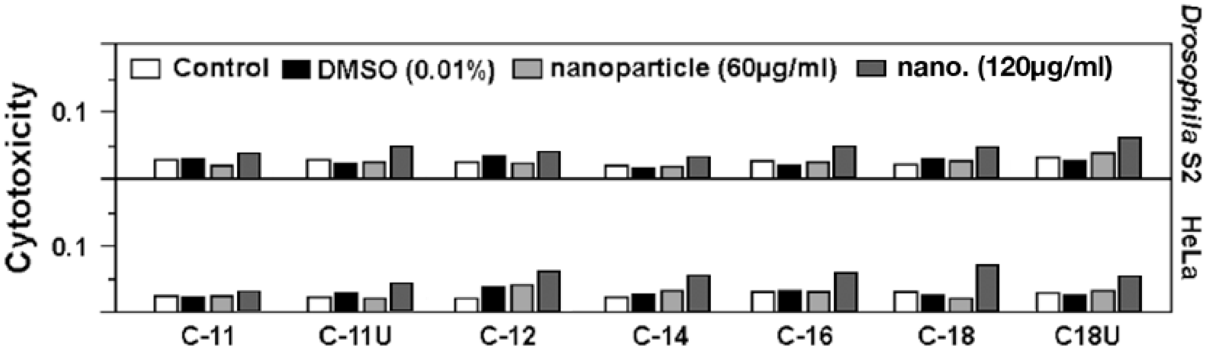

Figure S14. Cytotoxicity assay of *Drosophila* S2 and Human cancer cell lines (HeLa) incubated 3 days at various nanoparticles (60µg/ml) was performed. Cytotoxicity values correspond to the percentage of dead cells.

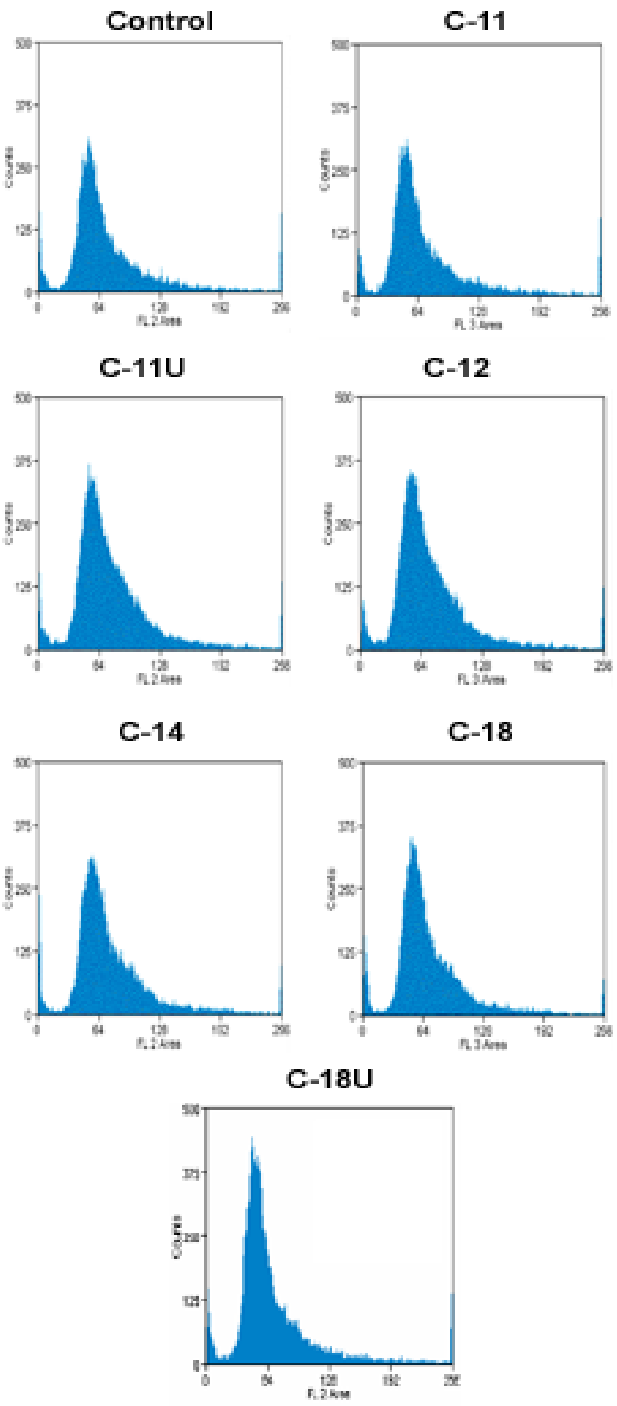

Figure S15. Flow-cytometry assay showing cell viability and cell cycle arrest profile for HEK293 cells incubated with various nanoparticles at a 60µg/ml concentration.

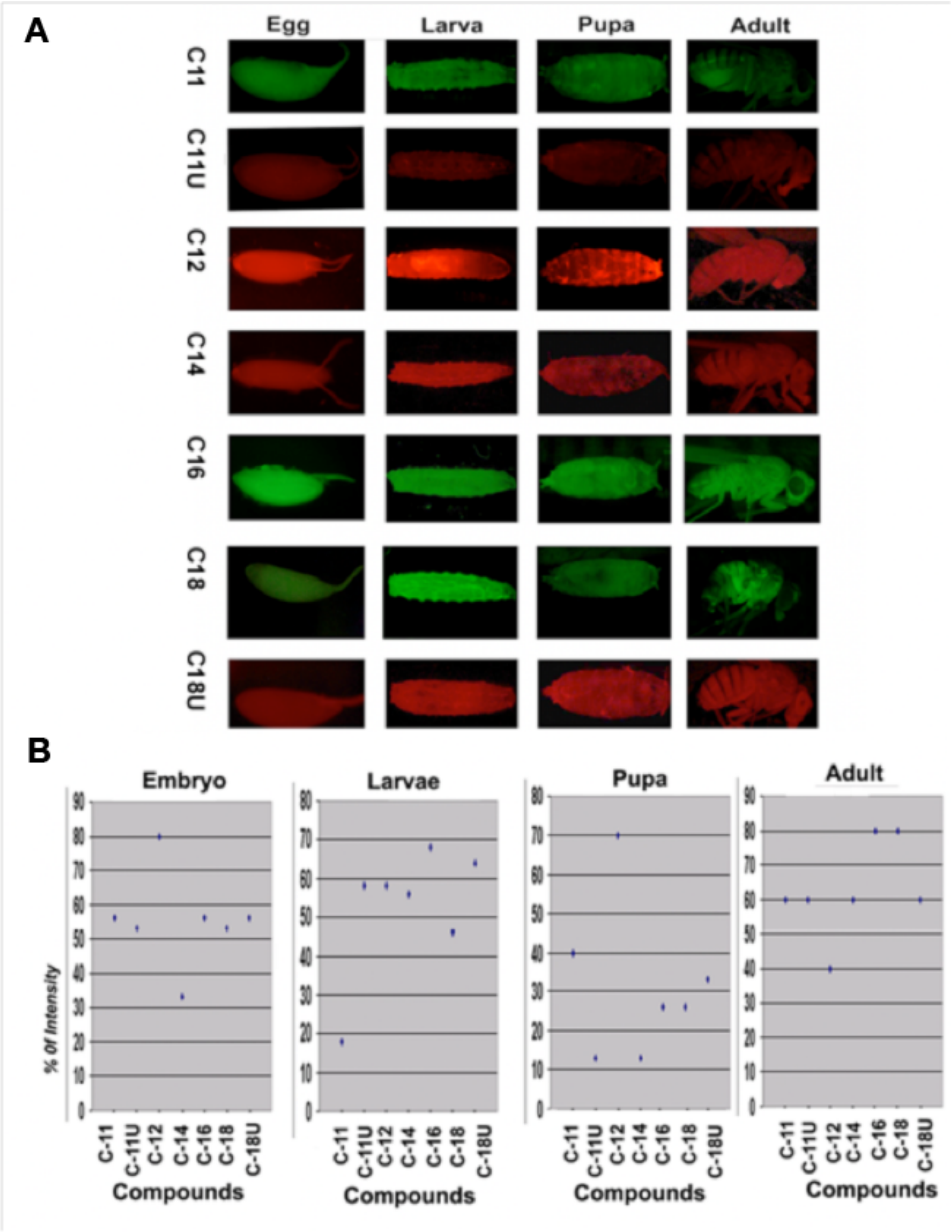

Figure S16. (A) Biocompatibility and distribution of nanoparticles in four different developmental stages of *Drosophila* progeny after feeding nanomaterials containing media of the parental population. (B) The amount of nanomaterials accumulation (proportional to the % of fluro-labelled intensity) were shown in the intensity maps. Each map was drawn by the mean values from five separate studies. The point refers the percentage of the accumulation of each nanoparticle in each developmental stage.

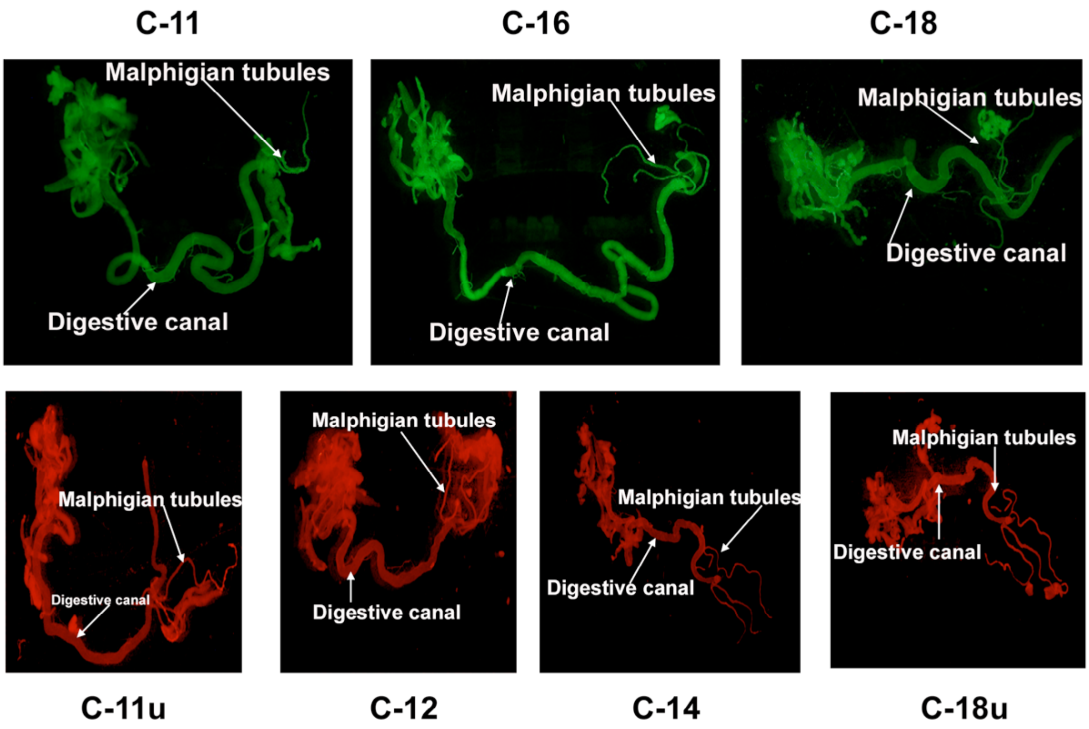

Figure S17. Distribution and spreading of seven nanoparticles in mouthpart and alimentary track and associated organs of *Drosophila* 3rd Instar larvae were shown. Different parts were marked with white arrows.

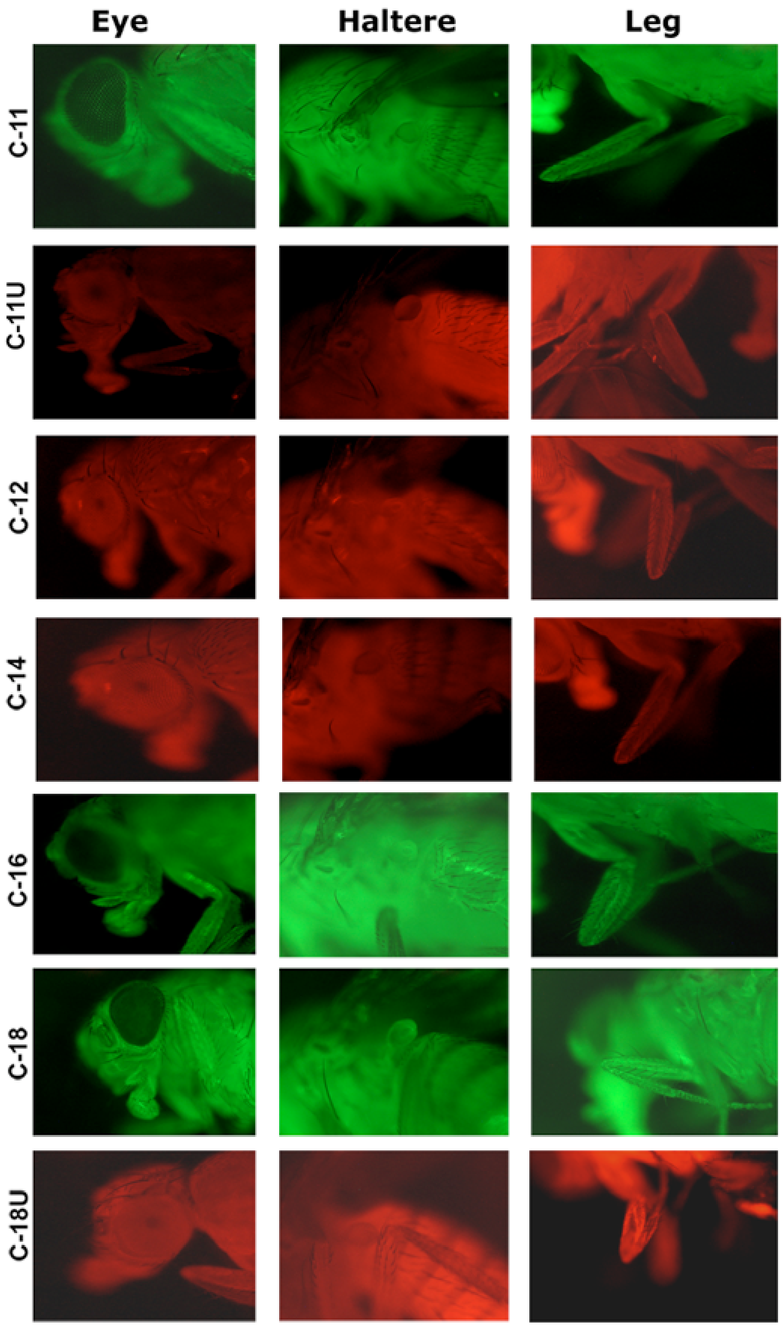

Figure S18. Delivery and spreading of seven nanoparticles in different adult organs of *Drosophila melanogaster* after feeding with yeast paste containing nanomaterials were shown.
